# Light-Driven Chemical Cascade Reduces Barriers to Hydrogen Production

**DOI:** 10.1021/jacs.5c07557

**Published:** 2025-06-20

**Authors:** Venugopala Rao Battula, Gabriel Mark, Muhammad Saad Naeem, Yash Shah, Michael Volokh, Leanne M. Gilbertson, Núria López, Menny Shalom

**Affiliations:** † Department of Chemistry and Ilse Katz Institute for Nanoscale Science and Technology, 26732 Ben-Gurion University of the Negev, Beer-Sheva 8410501, Israel; ‡ Institute of Chemical Research of Catalonia (ICIQ-CERCA), The Barcelona Institute of Science and Technology (BIST), Tarragona 43007, Spain; § Universitat Rovira i Virgili, Pl. Imperial Tarraco 1, Tarragona 43005, Spain; ∥ Department of Civil and Environmental Engineering, 3065 Duke University Durham, North Carolina 27708, United States

## Abstract

Photocatalysis offers an opportunity for sustainable hydrogen and chemical production. Traditional systems require semiconductors with very specific conduction-band (CB) properties and expensive noble metal cocatalysts, limiting material availability and increasing costs. Here, we introduce an alternative photocatalytic pathway that bypasses these constraints, producing hydrogen and formic acid via a cascade process. Under illumination, oxygen and methanol are converted to hydrogen peroxide and formaldehyde, which then react in solution to yield hydrogen and formic acid. We demonstrate the viability of the process employing two limited direct photocatalysts, polymeric carbon nitride (which is not active without a cocatalyst) and tungsten oxide (which presents an unsuitable CB). Our method provides significant advantages: bandgap flexibility, reduced energy consumption and environmental impact, and elimination of noble metal cocatalyst costs. This approach expands the range of suitable semiconductor materials for efficient photocatalytic hydrogen production, offering a more economical and practical solution.

## Graphical abstract

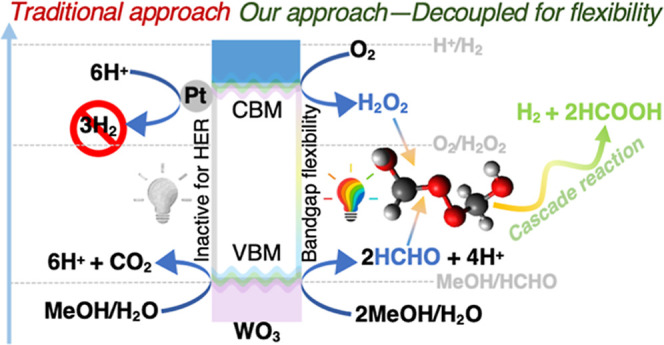

## Introduction

Photocatalysis offers an approach to produce clean hydrogen that is cost-effective and requires lower resource intensity compared with current alternatives. The common operation principles of photocatalysis require a material (usually a semiconductor) that, upon light irradiation, generates an electron–hole pair capable of splitting water by water reduction to hydrogen and oxidation to oxygen. In practice, several limitations complicate the photocatalytic event, impeding it. The foremost requirement is that the energy level positions of the photoabsorbing semiconductor align with water reduction (i.e., hydrogen evolution reaction, HER) and oxidation (i.e., oxygen evolution reaction, OER) potentials, demanding relativity negative and positive conduction (CB) and valence (VB) bands positions, respectively.^1^

As many photoactive materials are not capable of splitting water on their own, cocatalysts (usually up to ∼3% wt) are used to facilitate HER, OER, or both.^2^ The most efficient cocatalysts typically contain noble metals (e.g., Pt for HER and IrO_*x*_ for OER), which dramatically increase the cost of the catalytic system. Adding a cocatalyst usually requires further synthetic steps, and the overall system might be sensitive to poisoning and leaching during operation, reducing the durability of the overall system. Moreover, in most cases, sacrificial electron donors are used to address the sluggish kinetics of water oxidation to molecular oxygen.

To reduce the overall cost of an envisioned photocatalytic system, research on alternative oxidation reactions that yield valuable products (biomass derivatives, alcohols, amines, etc.) instead of low-value oxygen alongside the HER has gained significant attention.^3^ The integration of these two reactions may improve photocatalytic yields sufficiently to enable the concurrent production of hydrogen and other valuable chemicals.

A cascade photocatalytic reaction enables the synthesis of products through multiple sequential reactions, where the product of one reaction serves as a reactant of the next.^4–6^ The key advantage is that multiple transformations occur in one pot, making the process more efficient and more sustainable by reducing the number of separation steps. Photocatalytic H_2_O_2_ production through a two-electron oxygen reduction reaction (ORR) offers an alternative reduction reaction to the challenging HER.^7–11^ H_2_O_2_ production avoids the use of a cocatalyst, and it does not require a very negative CB, as HER does.^12^ However, H_2_O_2_ selectivity is low due to H_2_O_2_ decomposition and further reduction to water over time. Therefore, direct use of the generated H_2_O_2_ for different chemical reactions offers new possibilities for the synthesis of value-added chemicals.^6,13^

Here, we propose a new photocatalytic cascade reaction that employs low-cost materials to generate clean hydrogen, alleviating the limitations on the semiconductor’s energy band positions and eliminating the need for cocatalysts. We demonstrate the advantages of this new approach with two widely used photocatalysts: polymeric carbon nitride (CN) and tungsten oxide (WO_3_). The proposed cascade reaction demonstrates a very high atom economy and the reduction of waste, as well as providing a framework for efficient and economically viable chemical transformations across various systems.

## Results and Discussion

The new photocatalytic cascade concept is illustrated in Figure 1a. In this method, the photocatalyst simultaneously generates H_2_O_2_ via ORR and oxidizes methanol into formaldehyde (HCHO). In the second step of the cascade reaction, H_2_O_2_ reacts with HCHO, producing hydrogen and formic acid. To demonstrate the concept, we have chosen two abundant and cheap materials that are known for their inability to generate hydrogen for different reasons. Polymeric carbon nitride cannot generate hydrogen without a cocatalyst, while WO_3_ is an inactive photocatalyst for hydrogen production owing to an unsuitable CB edge.^14^ CN was prepared by calcination of a melamine–cyanuric acid supramolecular precursor. Full characterization of the prepared CN is provided in Figures S1–S3 in the Supporting Information. Bulk WO_3_ was prepared according to the published literature procedure,^15^ and its formation was confirmed by XRD and UV–vis absorption analysis (Figures S4–S5). The characterization of commercially purchased WO_3_ nanoparticles (NPs) can be found in the Supporting Information (Figures S6–S8).

**Figure 1:**
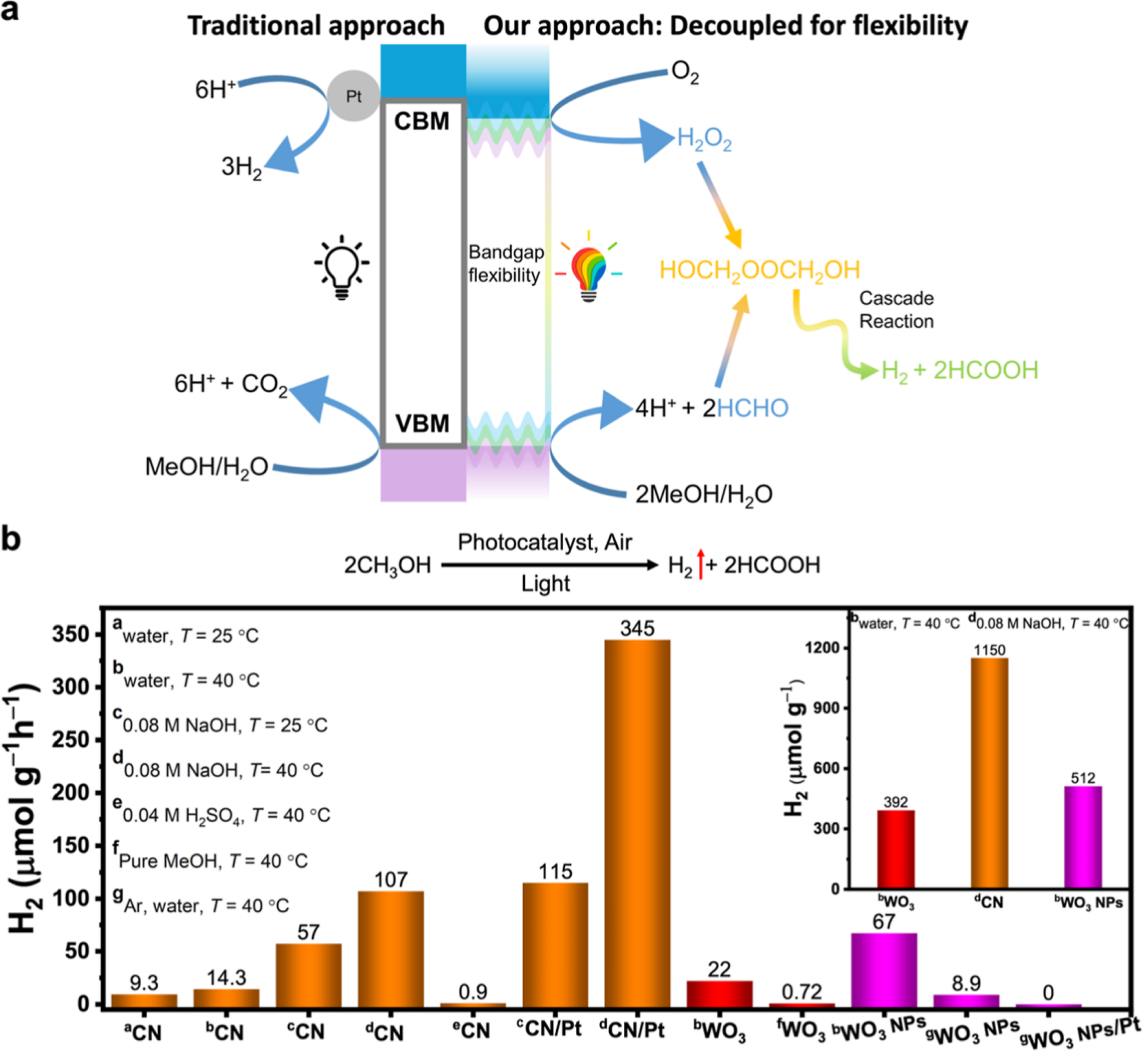
(a) Schematic diagram of the proposed H_2_O_2_-mediated cocatalyst-free H_2_ and HCOOH production over a photocatalyst in comparison with the traditional approach, which requires a Pt cocatalyst to be deposited before the reaction takes place. (b) Visible-light-driven cocatalyst-free H_2_ production over CN and WO_3_ photocatalysts. Comparison of average H_2_(g) production rate under different conditions. Reaction conditions: 20 mg catalyst, 5 mL of water (20% vol MeOH) or alkaline solution (20% vol MeOH, 0.08 M NaOH), static air, 100 W white LED irradiation ((7.9 ± 0.9) × 10^3^ W m^–2^; Figure S9b), 4 h reaction time. Inset: H_2_ production after 24 h.

To look for a potential cascade reaction, pairing up the rates at which each of the events takes place is of fundamental relevance. It is known that in solution, the reaction of H_2_O_2_ with HCHO is very fast and exclusively produces H_2_ and HCOOH without the need of a catalyst. Inspired by this observation, we aimed to design a photocatalytic system capable of concurrently producing H_2_O_2_ and HCHO. We selected polymeric CN as the photocatalyst based on its high H_2_O_2_ production yield by two-electron O_2_ reduction (ORR) under an ambient atmosphere. As for HCHO, it can be readily produced by the oxidation of methanol (MeOH). Thereafter, the in situ generated H_2_O_2_ and HCHO react to produce H_2_ and HCOOH without requiring a cocatalyst.

Initially, we studied and optimized the reaction parameters, such as solvent and reaction temperature, by performing photocatalysis experiments (experimental setup in Figure S9) under visible-light illumination (Figure 1b) and assessing average product rates. Over 4 h, CN produced 0.7 μmol H_2_ at a rate of 9.3 μmol h^–1^ g^–1^ from a solution of water/MeOH at 25 °C, and it produced 1.1 μmol H_2_ with a rate of 14.3 μmol h^–1^ g^–1^ at 40 °C. In an alkaline solution (0.08 M NaOH), CN produced more H_2_ (4.6 μmol) at a rate of 57 μmol h^–1^ g^–1^ at 25 °C; further increasing the reaction temperature to 40 °C resulted in the production of 8.5 μmol H_2_ at a rate of 107 μmol h^–1^ g^–1^. The same experiment in the presence of 0.04 M H_2_SO_4_ produced only 0.07 μmol H_2_ at a rate of 0.9 μmol h^–1^ g^–1^. Thus, we used 0.08 M NaOH and 40 °C as the optimized conditions for further studies using the CN photocatalyst. For comparative purposes, we performed the traditional photocatalytic HER experiments in 20% vol MeOH in 0.08 M NaOH using CN powder loaded with 3% wt Pt as the cocatalyst; over 4 h, 9.2 and 28 μmol H_2_ were produced at a rate of 115 and 345 μmol h^–1^ g^–1^ at 25 and 40 °C, respectively.

This approach allows the use of any H_2_O_2_-producing photocatalyst for H_2_ production, without requiring any cocatalyst(s). To demonstrate the advantage of this new H_2_ production process, we tested WO_3_, a visible-light photocatalyst known for its two-electron ORR activity, yet inactive in photocatalytic H_2_ production. Here, we directly tested at an optimized temperature (40 °C) in pure water (avoiding alkaline conditions as WO_3_ may react with NaOH to produce Na_2_WO_4_). Remarkably, during a 4 h experiment, bulk WO_3_ powder produced 1.8 μmol H_2_ at a rate of 22 μmol h^–1^ g^–1^ from water/MeOH at 40 °C. The same experiment in pure MeOH produced only 0.1 μmol H_2_ at a rate of 0.72 μmol h^–1^ g^–1^, which confirms the necessity of water. To verify the suggested mechanism and show the generality of our approach, commercial WO_3_ nanoparticles (NPs; 50–100 nm size) were tested. Before our MeOH photooxidation experiments, we tested the ability of WO_3_ NPs to produce H_2_O_2_ under light illumination in the presence of 20% vol ethanol (EtOH) in water at 40 °C; after 4 h illumination with visible light, iodide ions (I^–^) were added to the solution as an indicator; iodide reacts with H_2_O_2_ to form triiodide (I_3_^–^). The presence of I_3_^–^ was detected by UV–vis spectroscopy, confirming that H_2_O_2_ was indeed produced (Figure S10). In water/MeOH at 40 °C, over 4 h, WO_3_ NPs produced 5.3 μmol H_2_ at an average rate of 67 μmol h^–1^ g^–1^, which is higher than with bulk WO_3_ or CN under similar conditions.

A control experiment under O_2_-free conditions (Ar atmosphere) showed negligible H_2_ production, confirming the crucial role of O_2_ in generating H_2_O_2_, which subsequently reacts with HCHO to produce H_2_ under these experimental conditions. In addition, to prove our hypothesis that these WO_3_ NPs are inactive in HER photocatalysis and only produce H_2_ via the reported H_2_O_2_-mediated mechanism, we performed a regular photocatalytic HER experiment under Ar atmosphere by applying 3% wt Pt as a reduction cocatalyst. We did not detect H_2_ under these experimental conditions,^16^ despite multiple attempts. The inset in Figure 1b shows the production of H_2_ from both the CN and WO_3_ photocatalysts after 24 h. Gas chromatography (GC) analysis of the headspace indicates that O_2_ is consumed as H_2_ is produced during this reaction, demonstrating the ORR-coupled H_2_ production (Figure S11).

To understand the reaction between in situ generated H_2_O_2_ and HCHO to produce H_2_ and HCOOH, we performed a direct reaction of commercial H_2_O_2_ (200 μL, 30% wt) and HCHO (200 μL, 37% wt) in pure water or 0.1 M NaOH­(aq) but without a photocatalyst and in the dark (Figure S12). The direct mixture in pure water at 25 °C produced 4.73 μmol h^–1^ of H_2_ over 19 h. No H_2_ was produced from an aqueous solution of HCHO in the absence of H_2_O_2_ or mixture of H_2_O_2_ and HCOOH even after 24 h, confirming the role of H_2_O_2_ in the oxidation of HCHO to produce H_2_ and HCOOH.

The same reaction in NaOH­(aq) solution at 25 °C produced 85 μmol h^–1^ of H_2_, in line with the yield improvement observed with the CN photocatalyst in 0.08 M NaOH. The increase in activity might be due to the formation of the hydroperoxide anion (HOO^–^), which is a better nucleophile than H_2_O_2_.^17^ No H_2_ was produced from HCHO in the absence of H_2_O_2_ even after 24 h, which further rules out the reaction of HCHO with NaOH to give H_2_. Increasing the reaction temperature to 40 °C increased the rate of H_2_ production (to 99 μmol h^–1^), as was observed with CN in earlier experiments. HCHO alone produced H_2_ at a negligible rate (0.03 μmol h^–1^) at 40 °C, an almost 3300 times lower rate than with H_2_O_2_. This not only rules out the possibility of direct HCHO conversion to H_2_ but also proves the role of H_2_O_2_ in oxidizing HCHO to produce H_2_ under these experimental conditions.

To further understand the kinetics of the reaction of H_2_O_2_ with HCHO in pure water or 0.1 M NaOH­(aq), we monitored the H_2_O_2_/HOO^–^ UV–vis absorption of various solutions in situ in the dark at 25 °C for 1 h (Figures 2a,b and S13). Figure S13 shows no significant change in the individual UV–vis spectra of H_2_O_2_/HOO^–^ and HCHO over 1 h, showing that both compounds are stable in pure water and in 0.1 M NaOH. After mixing H_2_O_2_ and HCHO in pure water, the H_2_O_2_ band intensity slowly decreased during the 1 h experiment, which shows that H_2_O_2_ is being consumed by the reaction with HCHO (Figure 2a). Interestingly, the same mixture in 0.1 M NaOH showed a rapid weakening of the HOO^–^ UV–vis band (Figure 2b). The results of this in situ UV–vis analysis further demonstrate that the photocatalytic reaction of H_2_O_2_ and HCHO to yield H_2_ occurs more rapidly in 0.08 M NaOH (Figure 1b).

**Figure 2:**
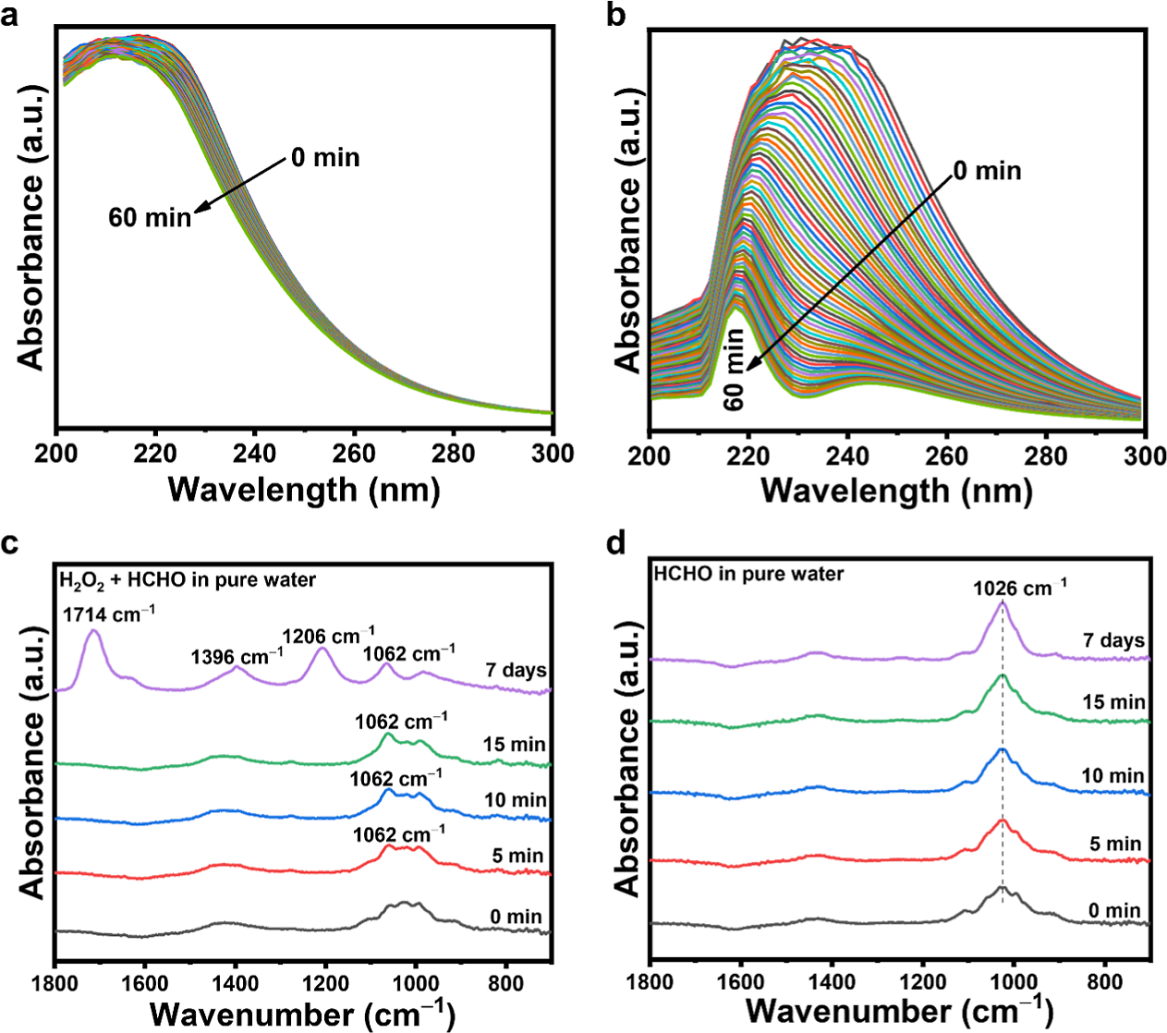
UV–vis absorption spectral change of a mixture of H_2_O_2_ and HCHO in (a) pure water and (b) 0.1 M NaOH­(aq) over 1 h. FTIR spectra of (c) a mixture of H_2_O_2_ and HCHO and (d) HCHO, both in pure water.

Because H_2_O_2_ reacts relatively slowly with HCHO in a neutral solution (Figure 2a), we hypothesized that it would be possible to observe the formation of new intermediate C–O bonds during the reaction using Fourier-transform infrared (FTIR) spectroscopy. Figure 2c shows FTIR spectra of a mixture of H_2_O_2_ and HCHO in water, where the peak at 1026 cm^–1^ corresponds to the C–OH group of the hydrated form of HCHO (methanediol) and gradually weakens during the measurement, while a new peak at 1062 cm^–1^, corresponding to C–O bonds, emerges. We ascribe this new peak to the intermediate HOCH_2_OOCH_2_OH. After 7 days, the C–OH group of hydrated HCHO had completely disappeared and new peaks had appeared at 1206 cm^–1^ and 1714 cm^–1^, which correspond to C–O and C=O stretching vibrations of HCOOH, respectively. In a control experiment, the FTIR spectrum of HCHO alone showed no change even after 7 days, and no formation of new C–O bonds was detected (Figure 2d).

To elucidate the critical intermediates involved in H_2_ production and the entire mechanistic pathway, we employed density functional theory (DFT-PBE) to compute the reaction energies for the catalytic and cascade reactions. The detailed reaction schemes for both reactions are illustrated in Figure 3a,b, respectively.

**Figure 3:**
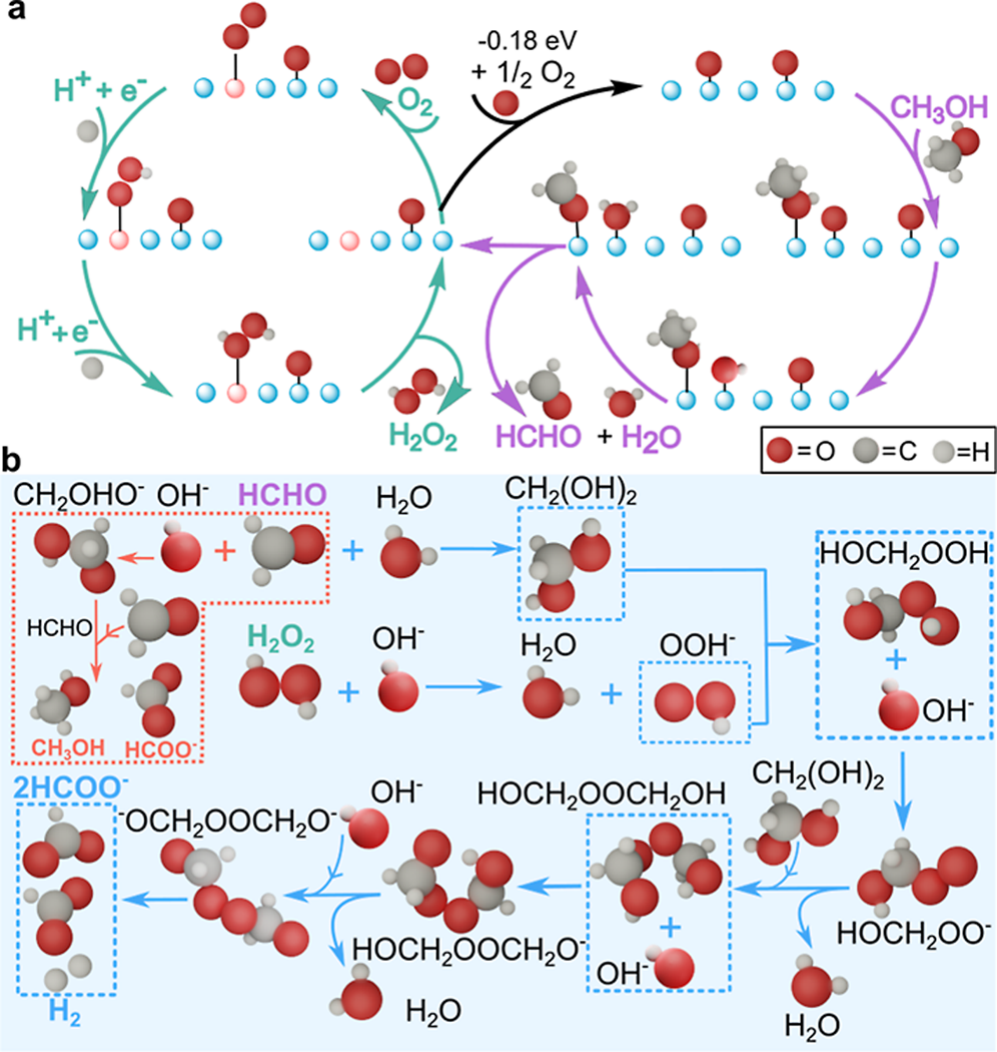
Reaction mechanisms for ORR, MeOH oxidation, and H_2_ and HCOOH production pathways as simulated at PBE-D3 DFT. (a) ORR (green) and MeOH oxidation (purple) mechanisms on WO_3_(001) interface. The MeOH oxidation encompasses CH_3_OH adsorption followed by two water-mediated proton transfers to the nearby surface oxygen, resulting in the formation of a water molecule that desorbs along with HCHO to form a surface oxygen vacancy (O_v_). ORR consists of O_2_ adsorption followed by two subsequent proton-coupled electron transfer (PCET) steps leading to the formation and desorption of H_2_O_2_. The transparent red sphere represents an oxygen vacancy resulting from methanol oxidation. The surface vacancy replenishment along with its energy is also illustrated. (b) Solution-phase homogeneous reaction mechanism for H_2_ production as proposed by Wieland and Wingler^18^ (blue) and the Cannizzaro pathway (red).^19,20^

The reactions of the cascade in which H_2_O_2_ reacts with HCHO to produce H_2_ and HCOOH are modeled without any catalyst via the mechanism proposed by Wieland and Wingler.^18^ They form HOCH_2_OOH (hydroxymethylhydroperoxide), an intermediate that further reacts with HCHO to form HOCH_2_OOCH_2_OH (bis­(hydroxymethyl)­peroxide), which finally decomposes to molecular hydrogen and formate (Figure 4b).^21^ Following a conformer search for the most stable HOCH_2_OOH and HOCH_2_OOCH_2_OH structures, the reaction energetics, intermediate vibrational frequencies, and a competing Cannizzaro pathway^19,20^ for formate and CH_3_OH production are presented in Figure 4b. The reaction energies for the formation of H_2_ and HCOOH and the competing Cannizzaro pathway are exothermic and confirm their thermodynamic feasibility. The C–O vibrational frequencies of the HOCH_2_OOH and HOCH_2_OOCH_2_OH intermediates (illustrated in Figure 4b) are 1031 cm^–1^ and 1054 cm^–1^, respectively. The theoretical frequency of HOCH_2_OOCH_2_OH (1054 cm^–1^) is in reasonable agreement with that experimentally observed at 1062 cm^–1^, validating the formation of the HOCH_2_OOCH_2_OH intermediate and its role in the reaction pathway toward H_2_ and HCOOH.

**Figure 4:**
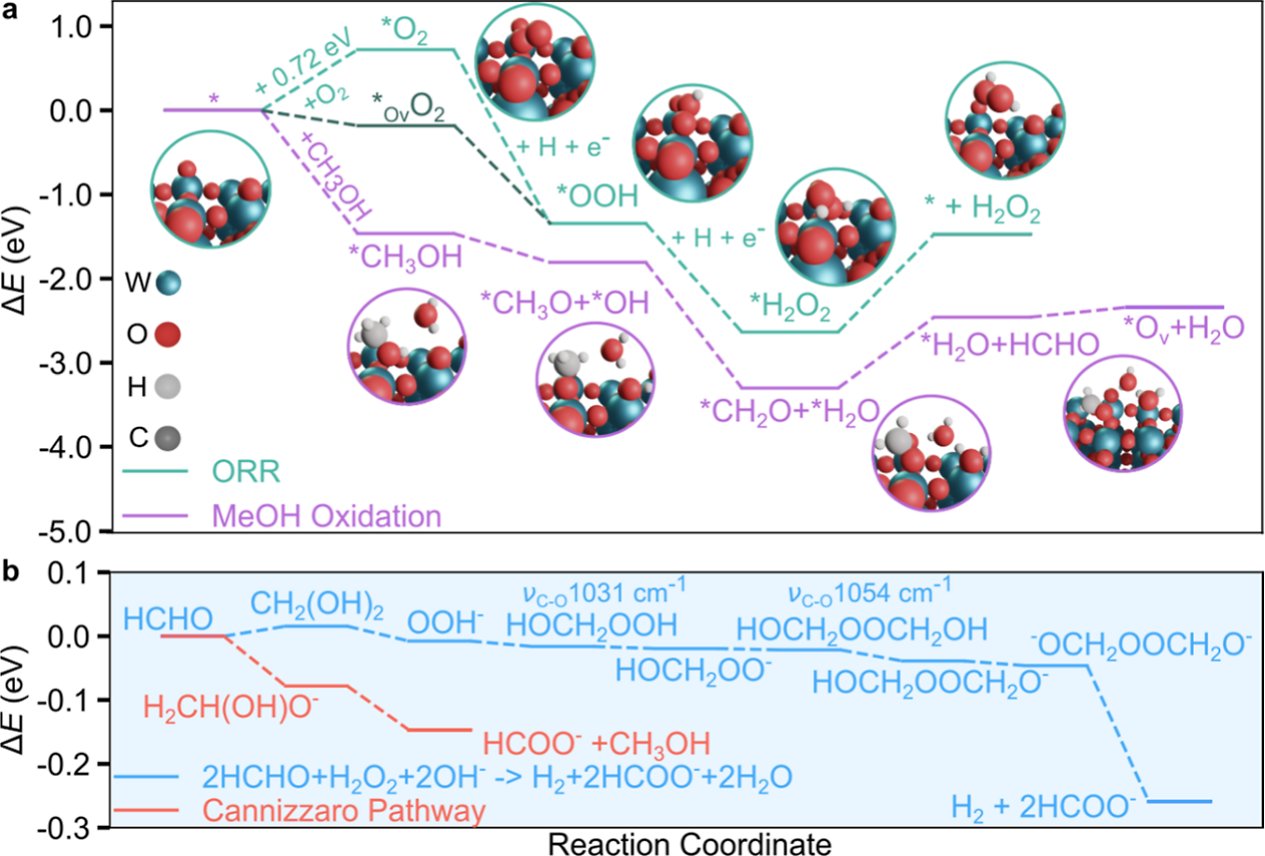
Reaction energetics for the ORR, methanol oxidation, and H_2_ and HCOOH production pathways. (a) ORR and methanol oxidation energetics on WO_3_(001) with reaction path illustrations as computed with DFT-PBE. MeOH oxidation is feasible because CH_3_OH adsorption and subsequent steps are exothermic. The O_2_ adsorption on the oxygen vacancy surface (*O_v_) formed during methanol oxidation is slightly exothermic, making ORR feasible. (b) Reaction energetics for H_2_ and HCOOH production from H_2_O_2_ and HCHO and the competing Cannizzaro mechanism.

The methanol oxidation reaction and ORR leading to H_2_O_2_ and HCHO were modeled on the WO_3_(001) surface. For methanol oxidation, the CH_3_OH adsorption and the subsequent steps are exothermic in the presence of an explicit water molecule acting as the proton transfer mediator (Figure 4a). For the ORR, O_2_ adsorption on the pristine surface is endothermic (0.72 eV), but the subsequent proton-coupled electron transfer (PCET) reaction steps are exothermic (Figure 4a). Instead, O_2_ adsorption on the oxygen vacancy site—formed during methanol oxidation—is weakly exothermic (−0.18 eV), making ORR feasible. The surface vacancy replenishment, also feasible, competes with ORR. The WO_3_(001) surface construction and the computational details are outlined in the Supporting Information (Computational Details section).

Based on the above analysis and literature reports,^9,22^ a possible mechanistic pathway for the photocatalytic cocatalyst-free H_2_O_2_-mediated H_2_ production is proposed in Figure 5a. Upon visible-light illumination, photogenerated holes and electrons form in the photocatalyst. The holes oxidize each MeOH to HCHO and 2H^+^. The formation of HCHO was confirmed colorimetrically utilizing a Nash reagent (Figure S14). Subsequently, the photogenerated electrons reduce O_2_ through a two-electron reduction path to produce H_2_O_2_. The generated H_2_O_2_ immediately reacts with HCHO to form a corresponding HOCH_2_OOH, which further reacts with another HCHO to produce HOCH_2_OOCH_2_OH.^22^ This HOCH_2_OOCH_2_OH rapidly converts to H_2_ and two molecules of HCOOH.

**Figure 5:**
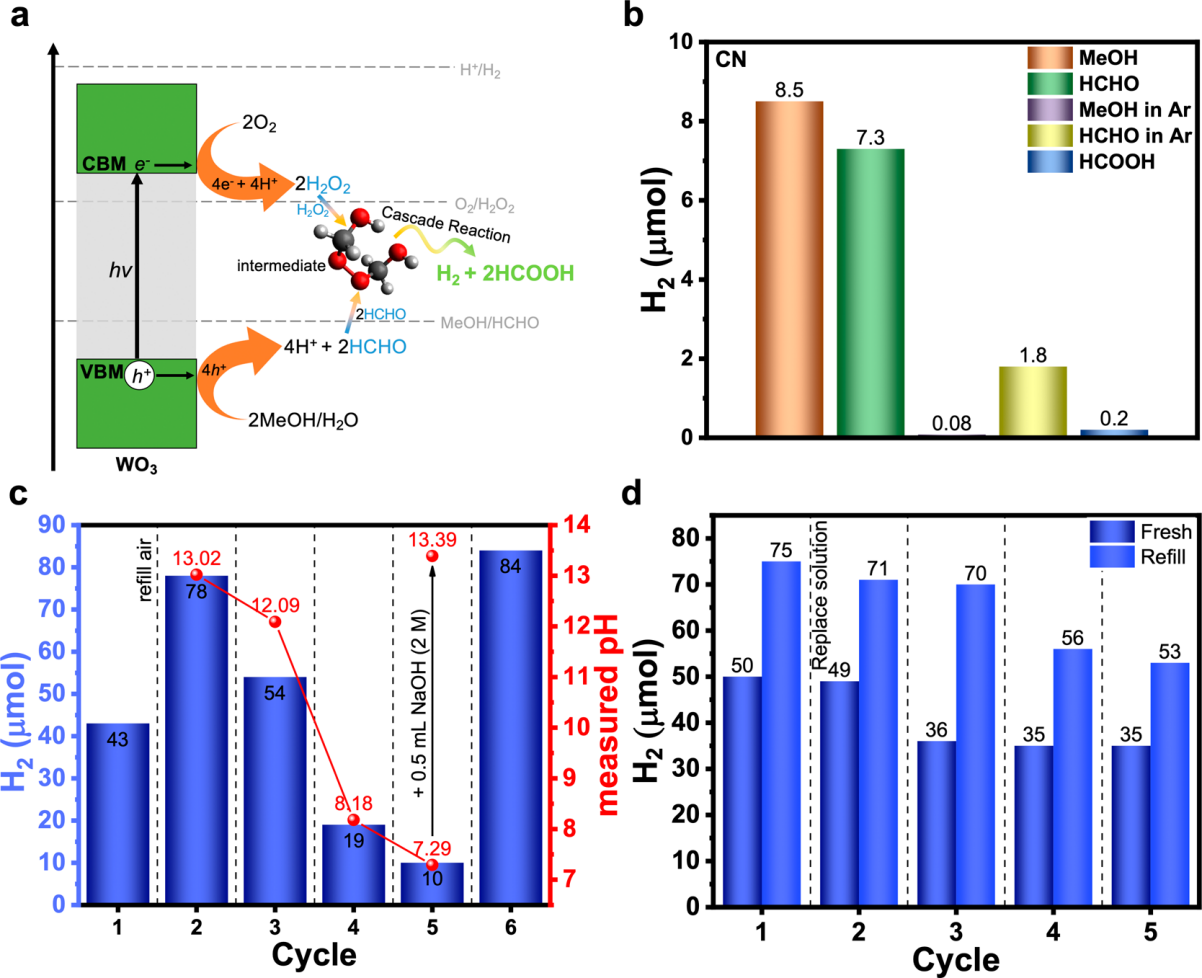
(a) Proposed mechanistic path for H_2_O_2_-mediated H_2_ production from MeOH oxidation over WO_3_, (b) comparison of H_2_ yields obtained under different conditions. Reaction conditions: 20 mg of CN photocatalyst, 5 mL 0.08 M NaOH aqueous solution, 20% vol of which is the reactant: (i) MeOH, (ii) HCHO (added as 1 mL of 37% wt in water; 10–15% MeOH stabilizer), or (iii) HCOOH, 20 mg CN, static air, 40 °C, 100 W white LED irradiation, ((7.9 ± 0.9) × 10^3^ W m^–2^). Cycle experiments: (c) for 6 days (24 h per cycle) and (d) for 10 days (48 h per cycle). Reaction conditions: 20 mg of CN photocatalyst, 5 mL of 20% vol of MeOH in 0.2 M NaOH, 20 mg CN, static air, 40 °C, 100 W white LED irradiation. In each cycle, after 24 h illumination, the solution was degassed, and the air was refilled before the subsequent 24 h illumination. The indicated pH values of the reaction mixture (including the catalyst) were recorded after each cycle.

To further support the proposed reaction pathway toward H_2_ and HCOOH, we quantified the amount of HCOOH obtained after 4 h using the CN photocatalyst. After 4 h, ∼23 μmol of HCOOH were produced (Figure S15a), exceeding the theoretically expected amount (17 μmol) as discussed in the supplementary discussion below Figure S15.

Under alkaline conditions at elevated temperatures, HCHO can undergo a Cannizzaro reaction (eq S3) to produce HCOOH and MeOH. Therefore, to evaluate the contribution of the Cannizzaro pathway under these experimental conditions, we performed a control experiment without a CN catalyst in the dark by directly monitoring 1 mL of HCHO (37% wt) in 4 mL 0.1 M NaOH at 40 °C. After 4 h, only a negligible amount of H_2_ (0.12 μmol) was produced, which is ∼71 times smaller than the amount produced by MeOH photooxidation using CN (8.5 μmol). HPLC analysis of the solution after the reaction showed the formation of HCOOH (Figure S15b). This suggests that the presence of an excess HCOOH after CN photocatalysis might be at least partially due to disproportionation of HCHO into HCOOH and MeOH through a Cannizzaro pathway, which does not contribute to H_2_ generation. Furthermore, proton nuclear magnetic resonance (^1^H NMR) analysis of the reaction mixture obtained after another photooxidation experiment using a CN photocatalyst also confirms the formation of HCOOH (Figure S16).

In addition, we performed control experiments with solutions containing either HCHO or HCOOH instead of MeOH in 0.08 M NaOH in the presence of the CN photocatalyst at 40 °C to understand the H_2_ production mechanism (Figure 5b). In a particular experiment, in the presence of HCHO, CN produced 7.3 μmol of H_2_ after 4 h, possibly due to the photooxidation of the 10–15% MeOH present in the HCHO solution as a stabilizer. After 24 h, the yield increased to 13 μmol only, a smaller amount than with MeOH (Figure S17). To further assess the possibility of direct photo-oxidation of HCHO to H_2_, we performed the same experiment under an Ar atmosphere. After 4 h, only 1.8 μmol of H_2_ was produced. This result confirms that the majority of H_2_ is being produced from the reaction between two photogenerated compounds, H_2_O_2_ and HCHO.

Moreover, the HCHO formed during MeOH photooxidation could have been expected to directly convert to HCOOH, which the CN photocatalyst (usually with a cocatalyst) could subsequently decompose to produce H_2_. To verify this hypothesis, we performed another control experiment using HCOOH instead of MeOH under the same photocatalytic experimental conditions. After 4 h, photoirradiation in the presence of CN produced only 0.2 μmol of H_2_, which is ∼42 times lower than with MeOH (8.5 μmol). After 24 h, the yield reached only 0.5 μmol (Figure S17). This observation strongly confirms that H_2_ is not produced by HCOOH decomposition.

Based on these observations (Figure 5b) and other previously discussed control experiments in the dark (Figure S12), we conclude that H_2_ is generated from the reaction of H_2_O_2_ and HCHO generated in situ via photocatalysis. Moreover, inspired by the recent increase in the significance of metal-free photocatalysts for environmental sustainability,^23^ and chemical transformations,^24^ we demonstrate a scaled-up photocatalytic reaction using 200 mg CN (Figure S18). It also enables the easy quantification of H_2_ produced in the dark after initial photocatalytic production of H_2_O_2_ and HCHO. The reaction can also occur in the presence of another base (K_2_CO_3_), with the yield depending on pH (see next section).

We performed 24 h cycle experiments (6 × 4 h cycle) using CN (Figure S19a), where after each cycle, the reaction mixture was degassed for 5 min under sonication and sealed with fresh air. The reaction mixture was then stirred in the dark for 15 min before light irradiation. From the second cycle onward, we detected a small amount of H_2_ produced at the beginning of each cycle after 15 min of stirring in the dark, which originates from the reaction of H_2_O_2_ with HCHO, both of which are left over from the previous cycle. This small H_2_ amount was subtracted from each cycle when estimating the H_2_ production under illumination. Overall, the 6 cycles (24 h; 4 h per cycle) yielded 57 μmol of H_2_, which is more than the 23 μmol generated in a single-cycle 24 h experiment (since O_2_ is consumed during the reaction and the air in the headspace was not replenished periodically in the latter case, oxygen serves as a limiting reagent). The increase in H_2_ yield in the initial cycles might stem from the reaction of H_2_O_2_ and HCHO left over from the previous cycle or the ability of leftover HCHO to act as a better hole scavenger than MeOH toward promoting an ORR.

Additionally, we performed another cycle experiment for 6 days (24 h per cycle) (Figure S19b). The H_2_ yield increased from the first to the second cycle and then decreased gradually in the next cycles. The upward and downward trends are similar to ones observed in the earlier 24 h cycle experiment. The significant decrease in H_2_ yield after the second cycle might be attributed to the decreasing concentration of MeOH; to test this hypothesis, before the fifth cycle, we added 1 mL of fresh MeOH to the solution. The H_2_ yield did not improve but continued to decrease. This decrease, even after the addition of MeOH, might result from the lowering the solution pH caused by the continuous consumption of OH^–^ over the 4 cycles. As shown earlier (Figures 3 and S12) the reaction between H_2_O_2_ and HCHO to form H_2_ and HCOOH occurs much more readily at high pH. Therefore, to evaluate the influence of pH, in the sixth cycle, we replaced the reaction solution with a fresh MeOH solution (20% vol MeOH in 0.08 M NaOH) and observed a dramatic increase in H_2_ yield. After 5 cycles of operation (5 days), CN retained ∼71% of its initial activity (Figure S19b). This shows the promising stability of CN for prolonged usage under these experimental conditions.

To further understand the influence of pH in the reaction, we performed another set of cycle experiments in 0.20 M NaOH for 6 days (24 h per cycle), in which we measured the pH of the reaction mixture after each cycle (Figure 5c). The reactions in 0.20 M NaOH exhibit similar trends to earlier experiments: the H_2_ yield decreases with decreasing solution pH. After the fifth cycle, the solution pH reached ∼7.3. In the sixth cycle, the pH of the reaction mixture was brought to ∼13.4 by adding 0.5 mL of 2 M NaOH, and H_2_ production increased dramatically. This shows the promising ability of CN to withstand prolonged use and maintain its performance as long as the solution pH is maintained.

Finally, we performed a 10-day recyclability experiment in 0.20 M NaOH solution, in which every 24 h, the air was replaced, and every 48 h, the solution was replaced (Figure 5d). The CN photocatalyst showed good stability throughout five cycles; the activity was slightly reduced after the second cycle, but it then remained stable over the next 3 cycles. After the fifth cycle, the catalyst was washed and dried (∼87.5% was recovered). Additionally, we have quantified the HCOOH after each 48 h cycle, showing a stable HCOOH production (Figure S20a,b). The recovered catalyst was analyzed by UV–vis and FTIR spectroscopy. The UV–vis and FTIR spectra of the CN after 10 days of reaction exhibit some minor changes compared to the fresh CN, which might be associated with the slight decline in activity after the second cycle (Figure S20c,d).

The photocatalytic method for hydrogen production presented in this research offers several advantages over conventional HER methods including, (i) the omission of a precious metal cocatalyst, (ii) the elimination of carbon dioxide coproduction, and (iii) the generation of a value-added product formic acid (compared with O_2_). Cocatalysts, such as platinum, have been employed with carbon nitride to enhance hydrogen production,^25–27^ yet such metals are associated with substantial environmental costs (i.e., environmental impacts, high resource demand) associated with their mining, extraction, and refinement processing.^28,29^ Furthermore, platinum is a finite resource and is categorized as a critical mineral, indicating an imbalance of its supply and demand. To further underscore the advantage of the presented cocatalyst-free CN system we conducted a life cycle impact assessment (LCIA) (see Tables S1–S4 for details on the inventory and uncertainty analysis, respectively).

The primary contributor to environmental impacts for the CN only and CN/Pt systems are electricity use during the synthesis. For the CN system, nitrogen used for providing inert atmosphere and cyanuric acid and melamine precursors also contribute substantially to the ozone depletion impact category (Figure 6a). For the CN/Pt system, the H_2_PtCl_6_ precursor contributes substantially (11–49%) to several of the impact categories (Figure 6b, ozone depletion, eutrophication potential, carcinogens, noncarcinogens, and ecotoxicity impacts), which emerges from extraction and processing of the platinum reagent. The assessment results highlight that energy demand (from electricity use) during synthesis is the predominant contributor to the environmental footprint in both material systems with >60% in all categories, except ozone depletion (OD), and as much as 99% for both CN and CN/Pt. Considerations on ways to reduce impacts from energy and N_2_ use, including the potential to adopt renewable energy sources and N_2_ recycling, are discussed in the Supporting Information (Note S1).

**Figure 6:**
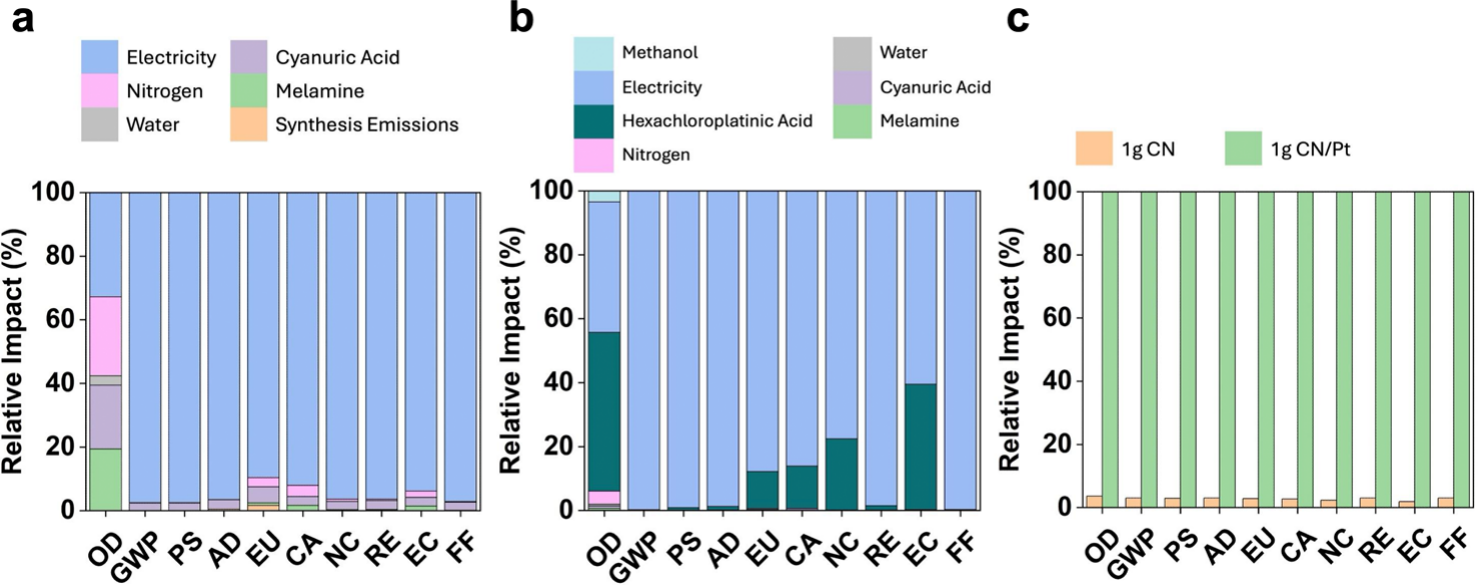
Process contributions associated with the ten tool for reduction and assessment of chemicals and other environmental impacts (TRACI) impact categories to produce 1 g of (a) CN, (b) CN/Pt, and (c) the relative impact assessment for synthesizing 1 g of CN and 1 g of CN/Pt. In (a), the primary contributors to environmental impacts are electricity and nitrogen. In contrast, (b) highlights that the majority of the impact of the CN/Pt synthesis stems from electricity and the production of hexachloroplatinic acid (H_2_PtCl_6_), the platinum precursor used for incorporating Pt into CN. (c) Further emphasizes the additional impact contributions associated with incorporating platinum in CN/Pt. The environmental impact of CN across all categories is, on average, ∼3% that of CN/Pt. In the figure, OD = ozone depletion, GWP = global warming potential, PS = photochemical smog, AD = acidification, EU = eutrophication, CA = carcinogenics, NC = noncarcinogenics, RE = respiratory effects, EC = ecotoxicity, FF = fossil fuel depletion.

A comparative analysis of the total synthesis impacts for the two material systems (Figure 6c) reveals that the relative environmental impacts of the CN/Pt synthesis far exceed that of the CN system presented herein (e.g., the global warming potential, GWP, for the synthesis of CN/Pt is more than × 34 that of the synthesis of CN; 39 and 1322 kg CO_2_ equiv per gram for CN and CN/Pt, respectively). An additional benefit of the CN-only photocatalytic system is the elimination of CO_2_ generation. Quantifying the associated CO_2_ equivalents provides a CO_2_ credit to the CN only system. Yet, at the current lab scale, the magnitude is quite low, 5.06 g CO_2_ equiv per 1 kg CN/Pt. Incorporation of this carbon credit is prudent with future scale-up.

Based on our rough cost estimations (as done in Note S2 and Table S5 from commercial vendors), the overall estimated catalyst cost using the reported new approach is more than × 5 times cheaper compared to conventional HER using CN/Pt for the same hydrogen average production rate of 1 L h^–1^. Moreover, a Pt cocatalyst is prone to CO poisoning or leaching from the catalyst surface, a problem which is completely ruled out in our proposed H_2_ production approach, facilitating scale-up for long-term application. Since recyclability is significantly easier for single-component systems, we propose that the potential lifetime of the Pt-free photocatalytic system can be enhanced by another order of magnitude, leading to a total of >15 times reduction in photocatalyst cost per L of produced H_2_. Additionally, this approach enables the use of other ORR active catalysts for H_2_ production; future studies may focus on developing low-cost photocatalysts with extended visible-light absorption to advance H_2_ production under natural sunlight without relying on a cocatalyst.

## Conclusions

We demonstrate a new photocatalytic route to produce H_2_ without the restrictions associated with the common photocatalytic path. Our approach enables H_2_ production by semiconductors inactive in HER (e.g., WO_3_), without the need for any cocatalyst, using only oxygen from air and MeOH. Under illumination, a cascade reaction occurs, whereby photogenerated H_2_O_2_ and HCHO further react, leading to H_2_ and formic acid formation. The reaction mechanism was confirmed by experimental and theoretical studies, verifying the reaction path and intermediates. Notably, the absence of cocatalysts (Pt) and the formation of a single valuable chemical in the aqueous phase (formic acid) significantly reduce the overall costs—not just the price tag but also the ecological footprint—of the catalytic system. This strategic cascade system paves the way toward application of photocatalytic systems that offer bandgap flexibility, reduce total energy consumption, and avoid the energy intensive liquid product separation. Furthermore, our approach increases photocatalyst stability and eliminates oxidative degradation. We envision that the described route would excite development of novel, thus far disregarded, “HER inactive” photocatalysts for H_2_ production.

## Supplementary Material



## Data Availability

DFT computational data in the ioChem-BD repository can be accessed following the link: http://dx.doi.org/10.19061/iochem-bd-1-384.

## Notes

Experimental details (catalyst synthesis, photocatalytic experiments, computational and life cycle impact assessment calculations), photocatalysts characterization, additional photocatalytic activity assessment: control experiments, products and intermediates identification and quantification, postoperation analysis, life cycle impact (LCI) assessment calculations, and cost estimation (PDF)
