# Supplementary material for: Light-Driven Chemical Cascade Reduces Barriers to Hydrogen Production
Source: J Am Chem Soc. 2025 Jun 20;147(30):26739–47. doi: 10.1021/jacs.5c07557 (PMC12314895; doi:10.1021/jacs.5c07557)
Supplement: Supplementary file 1 [file ja5c07557_si_001.pdf]

Light-Driven Chemical Cascade Reduces Barriers to Hydrogen Production

Venugopala Rao Battula[a], Gabriel Mark[a], Muhammad S. Naeem[b,c], Yash Shah[d], Michael Volokh[a], Leanne M. Gilbertson[d], Nuria Lopez[b]*, and Menny Shalom[a]**

[a]Department of Chemistry and Ilse Katz Institute for Nanoscale Science and Technology

Ben-Gurion University of the Negev

Beer-Sheva 8410501, Israel

E-mail: mennysh@bgu.ac.il

[b]Institute of Chemical Research of Catalonia (ICIQ-CERCA)

The Barcelona Institute of Science and Technology (BIST)

Tarragona 43007, Spain

E-mail: nlopez@iciq.es

[c]Universitat Rovira i Virgili, Pl. Imperial Tarraco 1, 43005, Tarragona, Spain

[d]Department of Civil and Environmental Engineering

Duke University

Durham, North Carolina 27708, United States

E-mail: leanne.gilbertson@duke.edu

Experimental Procedures: Materials and Characterization

Materials

All chemicals (reagents, solvents) were used as received from their commercial vendors without further purification.

Melamine (M, 99%), cyanuric acid (CA, 99%), 8% wt. chloroplatinic acid (H_2PtCl_6) solution, formaldehyde (37% wt. in H_2O; contains 10–15% MeOH as stabilizer), and WO_3 nanoparticles were purchased from Sigma-Aldrich. Hydrogen peroxide (H_2O_2, 30% wt.) was purchased from JT Baker. Potassium iodide (KI, ≥99%), ammonium acetate (≥99%), and formic acid (HCOOH, 98/100%) were purchased from Fisher Chemicals. Potassium hydrogen phthalate (KHT, HPLC grade) was purchased from Thermo Scientific. Ammonium paratungstate (99%) was purchased from Angene. Acetylacetone and acetic acid (99.7%, AR grade) were purchased from Lobachemie, India. Methanol (MeOH, 99.9%, absolute), ethanol (EtOH, 99.9%, absolute), Sulfuric acid (H_2SO_4, 95–98%), and acetone (AR grade) were purchased from Bio-Lab, Israel.

High-purity oxygen (99.99%) and argon (99.999%) gases were purchased from Maxima Ltd., Israel.

Deionized (DI) water for all aqueous solution preparations was collected after treatment through a Millipore Direct-Q 3 water purification system (resistivity of 18 MΩ cm at room temperature).

Characterization Techniques

Powder X-ray diffraction patterns (XRD) of CN and WO_3 were recorded using a PANalytical's Empyrean diffractometer, which is equipped with a position-sensitive detector X'Celerator (scan time of ~15 min, 2θ ranging from 5–20° to 60–90°, depending on the material, using Cu Kα radiation, λ = 1.54178 Å, 40 kV, 30 mA).

Nitrogen sorption measurements and specific surface area calculations using the Brunauer–Emmet–Teller (BET) model from the adsorption isotherm were performed on a Quantachrome NOVAtouch NT LX3 system at 77 K.

Fourier-transform infrared spectroscopy (FTIR) was performed to analyze the functional groups of CN and WO_3 using a Thermo Scientific Nicolet iS5 FTIR spectrometer (KBr window; diamond iD7 attenuated total reflectance (ATR) optical base; each measurement consisted of 16 scans with a 4 cm^{-1} resolution in the 400–4000 cm^{-1} range).

UV–vis absorption spectroscopy in reflectance (R) mode—using a diffuse reflectance accessory (DRA)—was recorded on a Cary 100 spectrophotometer; for simplicity, it is referred to as DRUV–vis (diffuse reflectance UV–vis). Tauc plot analysis was conducted (using a Kubelka–Munk analysis of the reflectance spectra) to estimate the optical band gap, assuming a direct band gap for CN and indirect band gap for WO_3.

The irradiance spectrum of the 100 W white LED (the visible range illumination source for photocatalysis, LZ-220V, LCFocus, China, driven using a constant 3 A current), incident was recorded using a StellarNet Black-Comet-C-SR-50 spectrometer measuring the irradiance through an F600-UVVIS-SR 600 μm single-strand fiber-optic cable ending with a CR2 cosine receptor and

a 2.7% aperture, all calibrated for irradiance using a NIST traceable lamp (StellarNet Inc., USA). The detector was placed at the lateral position of the reaction tube inside a water jacket (the difference being that the inner part of the reaction vessel was not filled with water, only the jacket). The incident energy flux of the white LED illumination source on the reaction tube was calculated by integrating the obtained irradiance spectra from 300 to 800 nm of three separate measurements yielding (7.9 ± 0.9) $\times 10^3$ W m^{-2} (irradiance onset ~400 nm).

X-ray photoelectron spectroscopy (XPS) data was recorded using an X-ray photoelectron spectrometer ESCALAB-Xi+ ultrahigh vacuum (4×10^{-10} bar) apparatus with an Al Kα X-ray source and a monochromator. The X-ray beam size was 500 μm and the survey spectra were recorded with a pass energy of 150 eV. The individual high-resolution spectra were recorded with a pass energy of 20 eV. All spectra were calibrated relative to a carbon C 1s peak positioned at 284.8 eV to correct the charging effects.

The liquid products of MeOH oxidation were analyzed using an HPLC instrument (Agilent 1260) coupled to a diode array detector (DAD) and equipped with a Hiplex-H column. A 0.05 M H_2SO_4 solution was used as the mobile phase. The yield of HCOOH was quantified using calibration curves obtained from known concentrations of HCOOH samples (Figure S15c and Figure S20a).

The generated $H_2(g)$ was collected from the headspace of the reactor using a gas-tight syringe and analyzed by gas chromatography (Agilent 7820 GC system equipped with a CP-molecular sieve 5A and PoraPLOT Q columns coupled to a thermal conductivity detector, TCD).

The pH of the reaction mixture (including the catalyst) was recorded using a Lab Star PH 111 pH meter.

Synthesis

Synthesis of MCA Precursors

Melamine (M) and cyanuric acid (CA) (1:1 mol ratio) powders (total mass of 0.5 g) were dissolved in 25 mL of DI water and shaken in a tube overnight using an automatic shaker at 300 rpm. The obtained MCA supramolecular assembly was centrifuged at 6000 rpm (4430 rcf) for 15 min, washed with DI water three times, and finally vacuum-dried at 50 °C overnight, ready to be used as a precursor for CN synthesis. Multiple batches were synthesized at the same time to get large amount of MCA precursor.

Synthesis of CN Photocatalysts

CN powders were prepared by calcination of MCA supramolecular assembly powders, serving as the CN precursor according to our previously reported procedure.[1] The MCA powder (~4.5 g) was placed in a rectangular ceramic crucible ($l \times w \times h$ = 6.9 × 6. 9 ×1 cm^3) covered with a lid. Subsequently, the crucible was placed in a tube furnace, heated to 120 °C within 1 h followed by heating to 530 °C with a heating rate of 5 °C min^{-1} and maintained at 530 °C for 4 h under a constant N_2 flow. After calcination, the sample was cooled to 50 °C within 100 min under N_2 flow, which yielded (~18%) melon-type CN powder. The obtained CN powder shows a BET specific surface area of 46.5 m^2 g^{-1}.

Synthesis of Bulk WO_3 Photocatalysts

Bulk WO_3 was synthesized according to a reported procedure.[2] Briefly, 3 g of ammonium paratungstate was placed in a ceramic crucible and heated to 500 °C with a heating rate of 5 °C min^{-1} and maintained at 500 °C for 4 h under static air. After calcination the sample cooled down to room temperature, naturally.

Commercially available WO_3 NPs were purchased from Sigma-Aldrich (Product No. 550086; nanopowder < 100 nm particle size via TEM).

Photocatalytic H_2 and HCOOH Production Over CN and WO_3 Photocatalysts

The photocatalytic experiments were carried out in a typical quartz cell under visible light in a controlled atmosphere (static air, O_2(g), or Ar(g)). In a typical experiment, 20 mg of the chosen photocatalyst (CN powder, bulk WO_3, or WO_3 NPs) was dispersed in a volume of 5 mL basic (or neutral) aqueous solution containing 20% vol. MeOH (1 mL MeOH + either 4 mL 0.1 M NaOH(aq), or 0.25 M NaOH, or pure DI water). We note that other bases can be used (e.g., K_2CO_3); however, the reaction rate is slower due to the lower pH (for example, using 0.08 M K_2CO_3 as the base (pK_a = 10.25), after 24 h using CN photocatalytst, produced 5.5 μmol of H_2, lower than 0.08 M NaOH (23 μmol) due to lower pH (initial ca. 11.6).

The quartz cell was sealed with a rubber septum under ambient air. The quartz cell was kept in a water bath maintained at a temperature of 25 or 40 °C using a water-circulating chiller to a double-walled water jacket (Figure S9a). A 100 W white LED, $(7.9\pm0.9)\times10^3$ W m^{-2} (measured in the 300–800 nm range; onset ~400 nm), was used as a light source (Figure S9b). At the end of the illumination experiment, 200 μL of gas sample was collected from the headspace of the reaction utilizing a gas-tight syringe to analyze gaseous products using gas chromatography (GC).

The liquid products were analyzed using high-performance liquid chromatography (HPLC). It should be noted that during CN photocatalytic experiments in NaOH(aq), the formed HCOOH might be in an anionic form initially. However, during prolonged recyclability experiments, we observed a decrease in pH (Figure 5c). For consistent quantification of liquid products, we have diluted the 10 μL of filtered reaction mixture to a total volume of 1 mL using 0.05 M H_2SO_4 solution and analyzed in HPLC using 100% 0.05 M H_2SO_4 solution as the mobile phase. Thus, we have identified and quantified the product as HCOOH only. Throughout the manuscript, we represent the product in its neutral form, HCOOH.

Computational Details

Density functional theory (DFT) simulations were conducted using Vienna Ab Initio Simulation Package (VASP)[3] version 5.4.4. The functional of choice was Perdew–Burke–Ernzerhof (PBE).[4,5] Core electrons were represented by Projector Augmented Wave pseudopotentials (PAW)[6] and valence electrons were expanded in plane waves with a kinetic energy cutoff of 450 eV. The electronic convergence was at least 10^{-5} eV and the force threshold for ionic convergence was set to 0.02 eV $Å^{-1}$ for geometrical optimization. Frequency calculations were performed using a step size of 0.015 Å and all the atoms except the target molecule were fixed. The explicit solvation calculations (water-mediated methanol oxidation) were carried out by placing four water molecules close the adsorbate/target molecule.

The monoclinic bulk WO_3 ($P12_1/c1$) was optimized, with *k*-point sampling of 4×4×4. Then, the WO_3 (002) surface slab was constructed as a supercell ($\sqrt{2}\times\sqrt{2}$)R45° with 4 layers (lateral size 15.43 Å, thickness 15.36 Å, vacuum ~15 Å) and the *k*-point sampling was reduced to Γ point. The MeOH oxidation and ORR were computed using DFT-PBE as explained above in VASP. The potential energy of H^+ was obtained from the computational hydrogen electrode (CHE) framework. The gas-phase O_2 was optimized with a spin polarization calculation in an asymmetric cell. Slab calculations included a 15 Å vacuum layer, the dipole correction was applied in the *z* direction, and two bottom layers were fixed during the relaxation.

The conformer sampling for $HOCH_2OOH$ and $HOCH_2OOCH_2OH$ was carried out using the CREST[7] (GFN2-xTB)[8] and the resulting structures and cascade reaction energetics were computed with DFT as implemented in Gaussian09.[9] The B3LYP[10–12] correlation functional and 6-31G (d) basis sets[13–15] were used for all atoms. The most stable conformers for both intermediates were considered for the reaction energetics.

The files for all the structures can be inspected through the ioChem-BD database at the following link:
https://iochem-bd.iciq.es/browse/review-collection/100/96976/571927baaa1aadafacf7e69d

Life Cycle Impact Assessment (LCIA)

Environmental Sustainability Assessment of the Synthetic Procedures

Scope, System Boundary, and Functional Unit:
A cradle-to-gate LCIA was performed for two synthesis pathways: (i) polymeric carbon nitride (CN) and (ii) polymeric carbon nitride incorporating platinum (CN/Pt). This assessment encompasses all inputs, emissions, transportation, and processes involved, from raw material acquisition to the production of CN and CN/Pt, including upstream resource extraction, energy consumption for reagent production, and direct emissions from energy generation. A mass-based functional unit of one gram of synthesized CN or CN/Pt was selected to demonstrate the environmental impact reduction associated with the elimination of the Pt co-catalyst.

Impact Assessment:

The LCIA was conducted on the synthesis routes for the production of 1 g CN or CN/Pt described above (refer to Tables S1 and S2 for the inventory details of each synthesis). The impact assessment was performed using SimaPro 9.6.0.2 (PRé Consultants). The United States Life Cycle Inventory (USLCI)[16] was chosen when available; otherwise, Industry Data 2.0[17] or Ecoinvent 3[18,19] was selected. Environmental impacts were modeled using the United States Environmental Protection Agency's Tool for the Reduction and Assessment of Chemical and Other Environmental Impacts (TRACI) 2.1 assessment method.[20] Environmental impact categories include ozone depletion (OD, in kg CFC-11 eq), global warming potential (GWP, in kg CO_2 eq), photochemical smog formation (PS, in kg O_3 eq), acidification (AC, kg SO_2 eq), eutrophication (EU, kg N eq), human health impacts from toxic carcinogenic (HHC) and noncarcinogenic (HHNC) substances (in comparative toxic units for human toxicity impacts, or CTUh), respiratory effects (RE, in kg PM2.5 eq), ecotoxicity (EC, in comparative toxic units for aquatic ecotoxicity impacts, or CTUe), and fossil fuel depletion (FF, in MJ surplus); eq represents equivalents. Bare and colleagues provide a detailed overview of the impact-assessment methodologies and the characterization factors applied to each of the aforementioned TRACI impact categories.[21]

Uncertainty Analysis:

Uncertainty analysis was performed using Monte Carlo simulation (1000 runs, confidence interval 95%, SimaPro 9.6.0.) to assess the uncertainty of the cumulative unit process life cycle inventory data associated with each synthesis route. For unit processes available in the selected databases (i.e., USLCI, Ecoinvent, Industry Data 2.0), the default lognormal distributions were used. For the unit processes created by the authors, the uncertainty factors were calculated for each input and output data considering a lognormal distribution and utilizing the pedigree matrix approach (the factors used in the pedigree matrix are listed in Tables S1–S2, and the resulting standard deviations from the detailed uncertainty analysis are reported in Tables S3–S4).

Synthesis of 0.81 g CN (reaction yield of 18% wt.)—LCI:

LCI Inputs:

i. 2.23 g melamine
ii. 2.27 g cyanuric acid
iii. 225 mL of deionized water
iv. Nitrogen gas: assuming flow rate of 160 mL min^{-1} for the entire calcination process of (142 + 240 + 100 = 482 min) = 160 mL min^{-1} × 1.16 kg m^{-3} × 1 $m^3/10^3$ L = 0.089 kg
v. Electricity:
   a) Shaker (Heidolph, Unimax 1010): 50 W for 12 h
   b) Centrifuge (HERMLE, Z 206 A): 100 W for 15 min
   c) Vacuum Oven (SHEL LAB): 439 W at 150 °C for 12 h
   d) Vacuum Pump (Vacuubrand, MZ 2C NT): 180 W for 12 h
   e) Furnace (NBD-O1200-80IT): 4000 W for 8.03 h (note: this is maximal power, serving as an upper limit)

Total electricity consumption = (50 × 12) + (100 × 0.25) + (439 × 12) + (180 × 12) + (4000 × 8.03) = 40.173 kWh

vi. LCI emission to air:
Ammonia 0.60 g
Isocyanic acid 2.27 g
Water 225 g
Nitrogen 89 g

Synthesis of 20 mg CN/Pt (Pt yield of 100% wt.)—LCI:

LCI Inputs:

i. 0.055 g melamine
ii. 0.056 g cyanuric acid
iii. 9.5 mL of deionized water
iv. 1.26 mg hexachloroplatinic acid
v. Nitrogen gas: Since 20 mg of CN was used in the synthesis, the nitrogen consumption was calculated proportionally to this mass, based on previous calculations for CN. The resulting nitrogen consumption was determined to be 9.88 g.
vi. 0.791 g methanol
vii. Electricity:
   a) 100 W LED (LZ 220 V): 100 W for 24 h
   b) Chiller (vivo RT4): 825 W for 24 h
   c) Stirrer (Heidolph, MR Hei-Tec): 300 W for 24 h

   Subtotal electricity consumption = (100 × 24) + (825 × 24) + (300 × 24) = 29.4 kWh
   Additional electricity consumption for 20 mg CN added to this process = 4.46 kWh
   Thus, the total electricity consumption = 29.4 + 4.46 = 33.86 kWh
viii. LCI emission to air:
Ammonia 0.01485 g
Isocyanic acid 0.056 g
Water 29 g
Nitrogen 9.88 g

Supporting Information Figures, Tables, and Notes

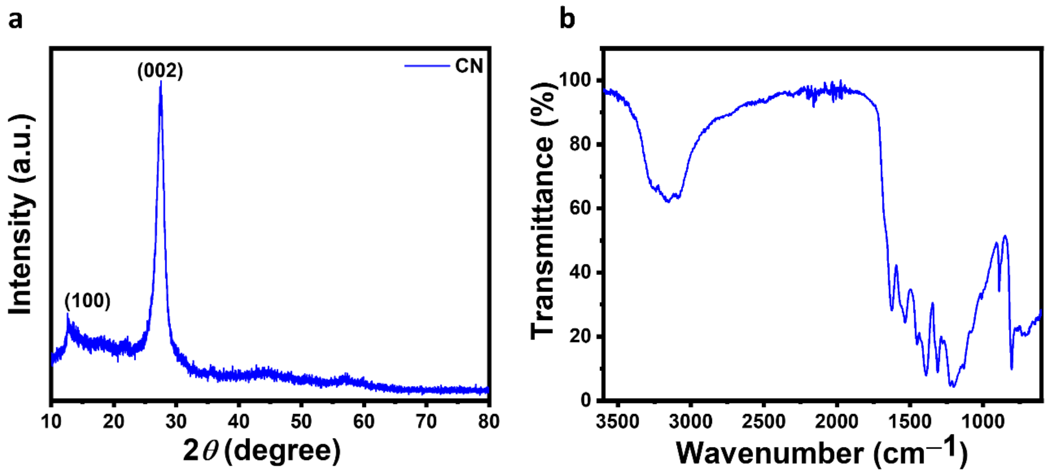

Figure S1. (a) XRD pattern and (b) FTIR spectrum of CN.

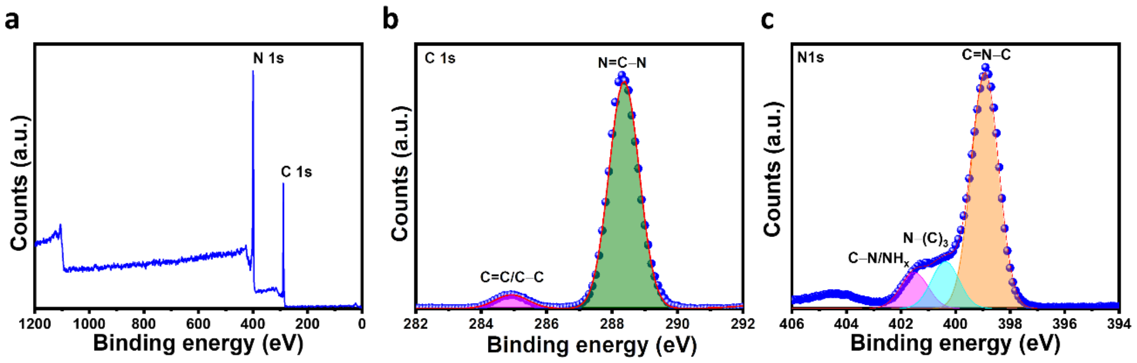

Figure S2. CN XPS analysis. (a) Survey scan; high-resolution (b) C 1s and (c) N 1s spectra.

The XRD presents diffraction signals around 2θ = 27.4° and 13.0°, which are assigned to the interlayer stacking of CN layers (002 planes) and in-plane ordering of the tri-*s*-triazine structure (100 planes), respectively (Figure S1a). The FTIR spectrum of CN presents characteristic peaks around 3200–3500 cm^{-1}, 1200–1650 cm^{-1}, and 804 cm^{-1}, which are assigned to –NH_x groups, C–N heterocycles, and heptazine/triazine moiety, respectively (Figure S1b). The XPS survey spectra of CN confirms the presence of C and N elements (Figure S2a). The individual high-resolution C 1s and N 1s spectra further confirm the nature of C and N in the CN sample.

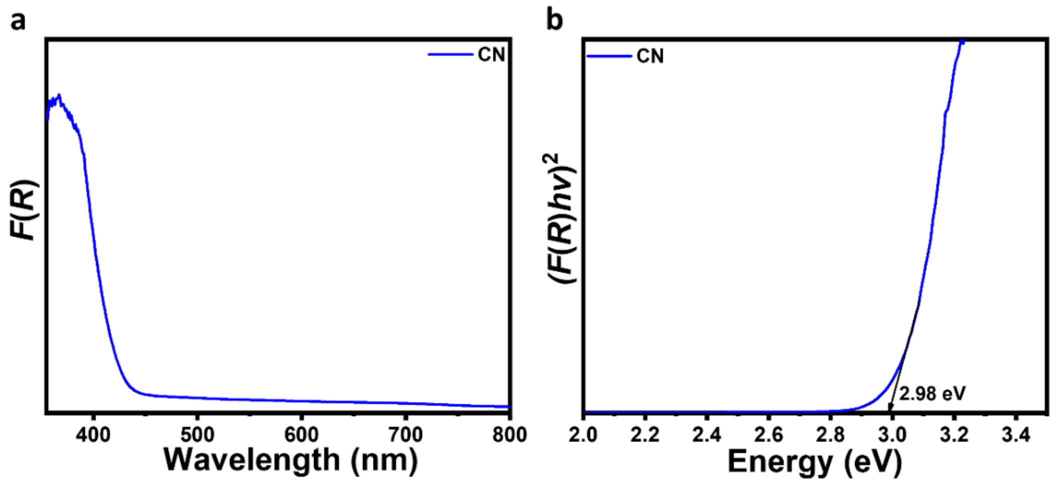

Figure S3. Optical absorbance characterization of CN. (a) DRUV–vis (shown as the *F*(*R*), proportional to logarithmic absorbance). (b) Tauc plot analysis assuming a direct optical band gap.

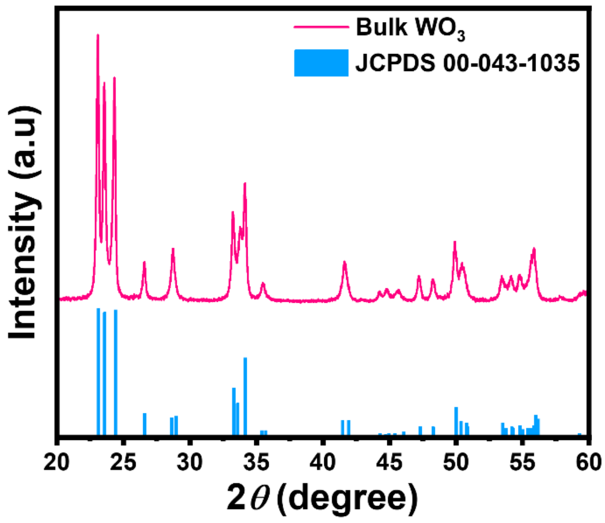

Figure S4. An XRD pattern of bulk WO_3 matching with standard patterns (JCPDS PDF no. 00-43-1035).

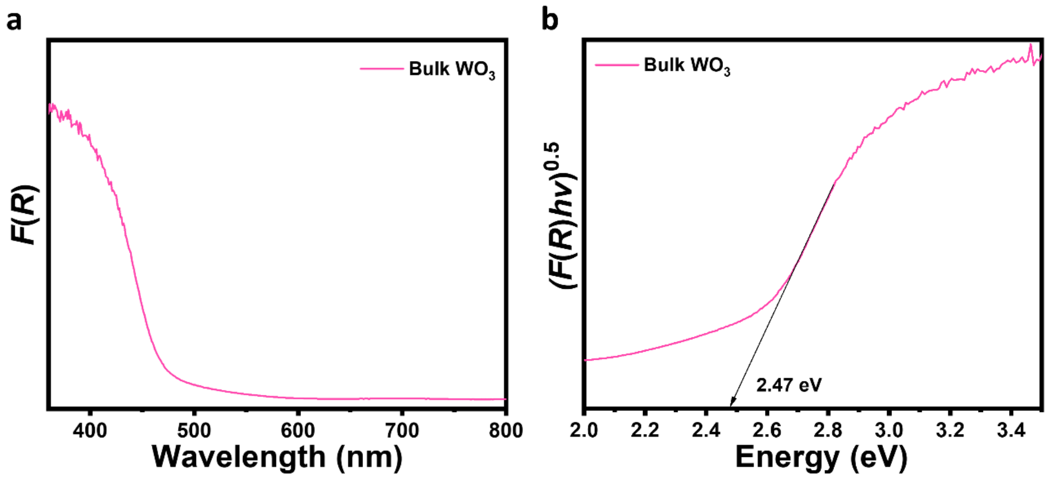

Figure S5. Optical absorbance characterization of bulk WO_3. (a) DRUV–vis (shown as the *F*(*R*), proportional to logarithmic absorbance). (b) Tauc plot analysis assuming an indirect optical band gap.

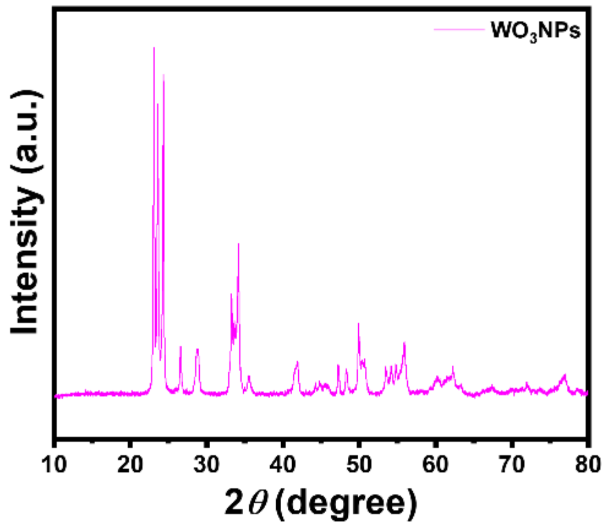

Figure S6. XRD pattern of WO_3 NPs.

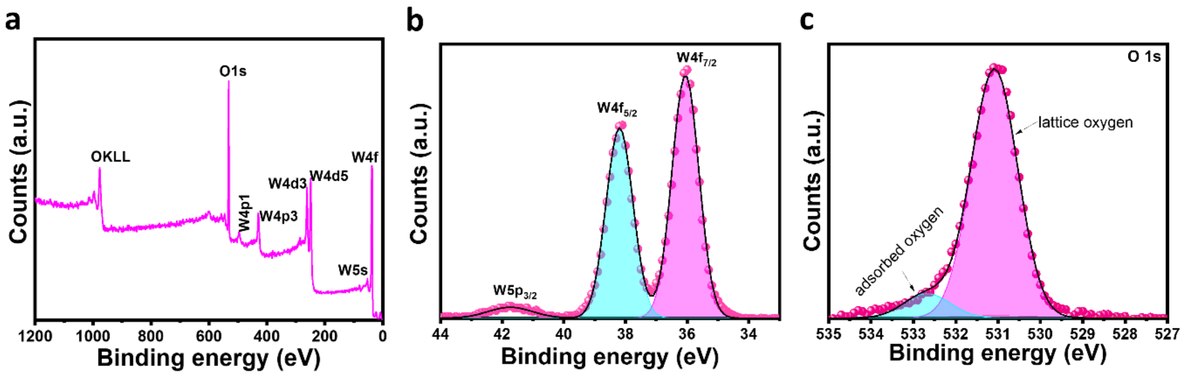

Figure S7. WO_3 NPs XPS analysis. (a) Survey scan; high-resolution (b) W 4f and W 5p, and (c) O 1s spectra.

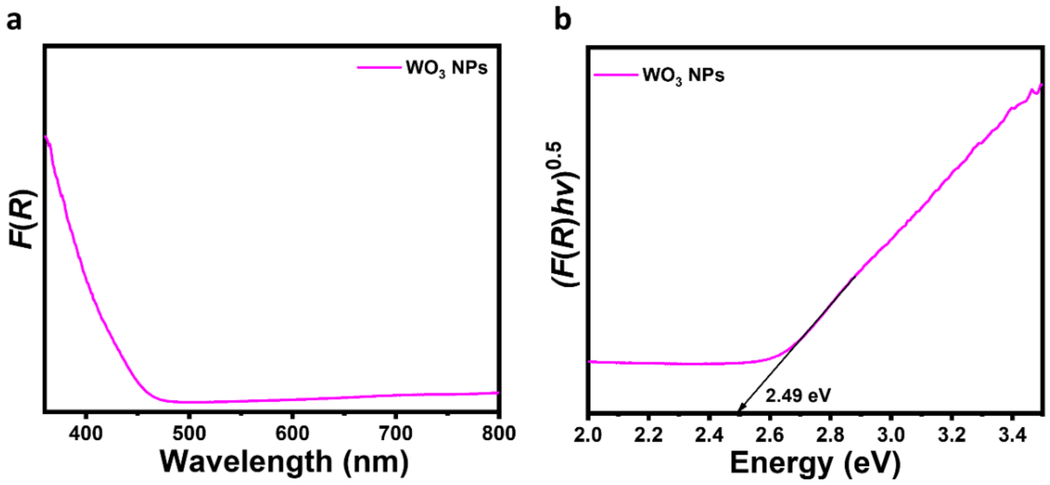

Figure S8. Optical absorbance characterization of WO_3 NPs. (a) DRUV–vis (shown as the $F(R)$, proportional to logarithmic absorbance). (b) Tauc plot analysis assuming an indirect optical band gap.

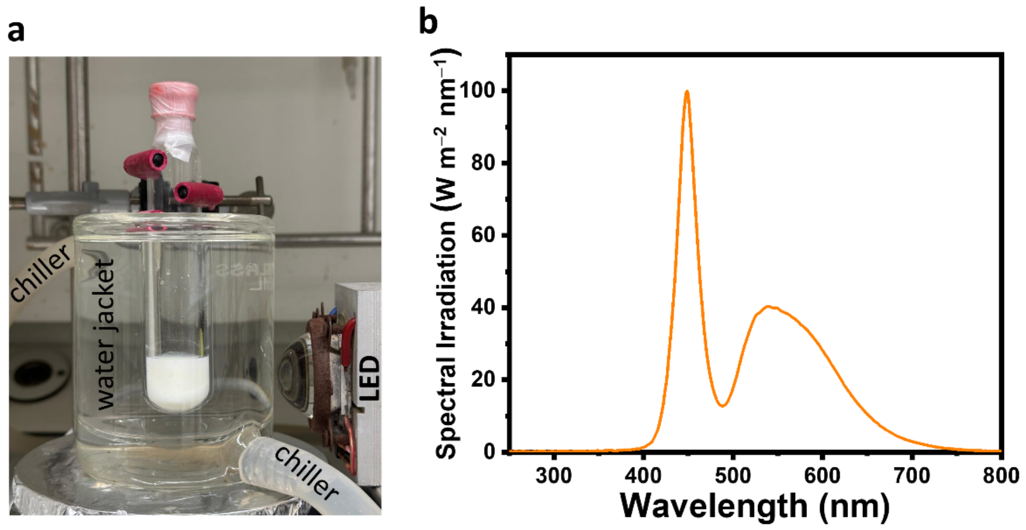

Figure S9. (a) The photocatalytic setup used for photocatalytic experiments comprising a quartz reaction tube, a double-walled jacket connected to a chiller offering temperature control (the inside is also filled with water to allow good heat transport) and an illumination source at a fixed position. (b) 100 W white LED irradiance spectrum used for photocatalytic experiments (measured with an optical fiber, connected to a spectrometer, placed instead of the quartz tube; the coolant water within the jacket's double wall was filled with water, but the vessel itself was empty to allow measurement; The average of three separate measurements gave $(7.9 \pm 0.9) \times 10^3$ W m^{-2}, integrated over a 300–800 nm range; the irradiance spectrum of a single measurement, closest to the average value is shown (7.01×10^3 W m^{-2}), the source onset in the visible range is ca. 400 nm.

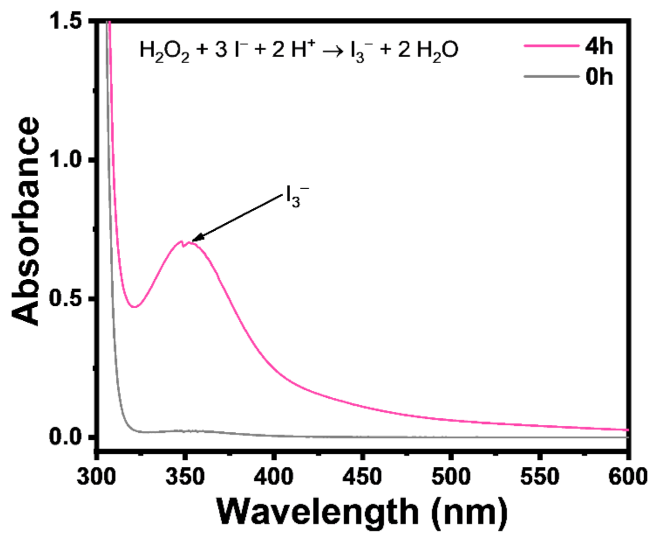

Figure S10. UV–vis spectra showing the formation of H_2O_2. Reaction conditions: 20 mg of WO_3 NPs photocatalyst, 5 mL of 20% EtOH, 40 °C, static air, 100 W white LED irradiation.

After 4 h experiment, 100 μL of the filtered reaction mixture was added to a mixture of 450 μL of 0.4 M KI and 450 μL of 0.1 M KHT solution. The ensuing solution was kept the in the dark for 30 min to allow a complete reaction of H_2O_2 with I^- under acidic conditions to form I_3^-, which gives a characteristic UV absorption peak at 350 nm as shown in Figure S10.

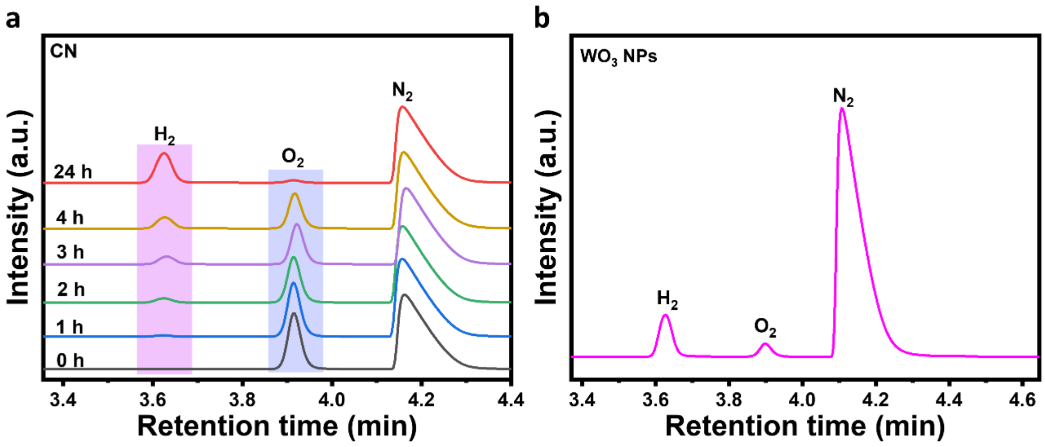

Figure S11. GC chromatograms of the gaseous products of the reaction over (a) CN, measured at different reaction times (0–24 h), and (b) WO_3 NPs, after 24 h.

Figure S11a shows the GC chromatograms measured during the MeOH oxidation experiment using the CN photocatalyst. It shows the gradual decrease of O_2 from air and a concurrent increase of H_2 during the photocatalytic MeOH oxidation reaction.

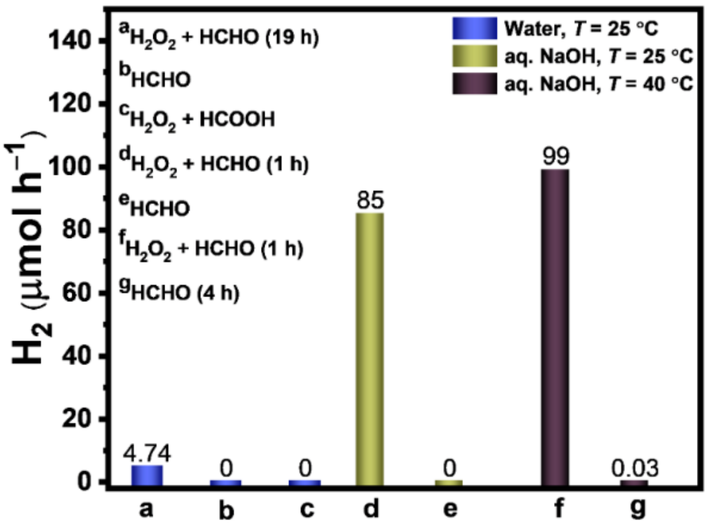

Figure S12. Control experiments in the dark in static air. Reaction conditions: (a) 200 µL of HCHO (37% wt.) and 200 µL of H_2O_2 (30% wt.) added to 4.6 mL of water, (b) 200 µL of HCHO (37% wt.) added to 4.8 mL of water, (c) 200 µL of H_2O_2 (30% wt.) and 200 µL of HCOOH (98/100% wt.) added to 4.6 mL of water, (d) 200 µL of HCHO (37% wt.) and 200 µL of H_2O_2 (30% wt.) added to 4.6 mL of 0.1 M NaOH(aq), (e) 200 µL of HCHO (37% wt.) added to 4.8 mL of 0.1 M NaOH(aq), (f) 200 µL of HCHO (37% wt.) and 200 µL of H_2O_2 (30% wt.) added to 4.6 mL of 0.1 M NaOH(aq) and (g) 1 mL of HCHO (37% wt.) added to 4 mL of 0.1 M NaOH(aq).

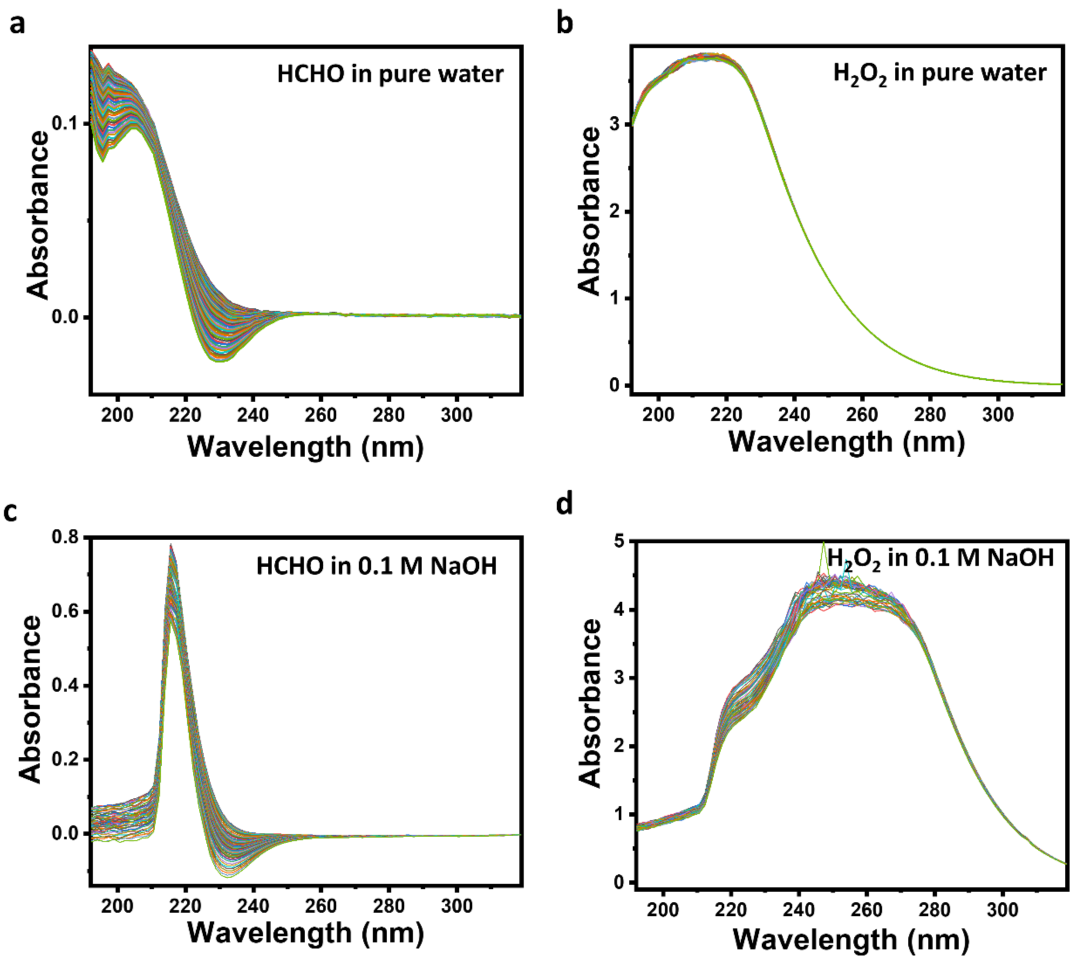

Figure S13. UV–vis absorption spectral change of (a) HCHO and (b) H_2O_2, both in pure water, and (c) HCHO and (d) H_2O_2 in 0.1 M NaOH for 1 h. The UV–vis absorption spectra were recorded from 190 to 800 nm in cycle mode with cycle count of 60 and cycle time of 1 min.

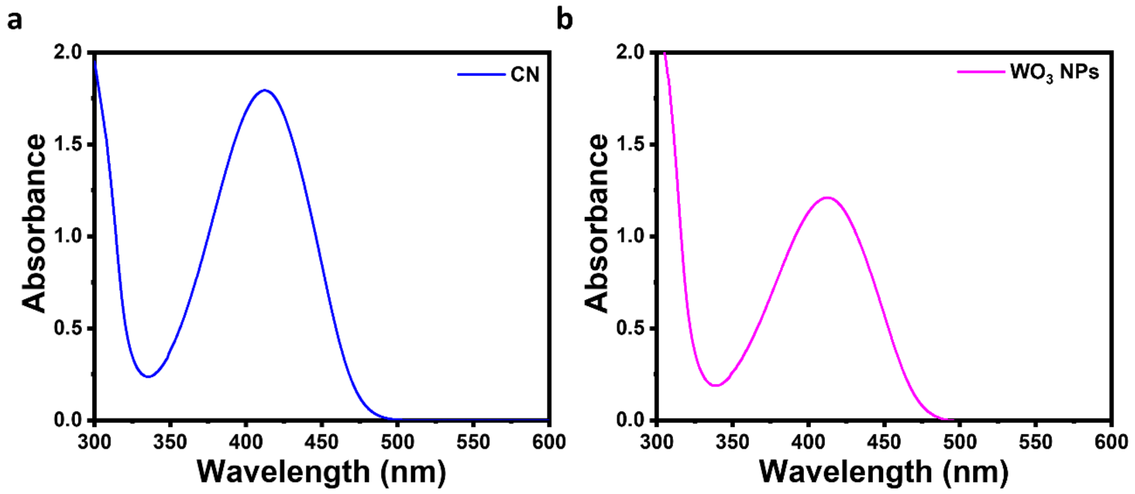

Figure S14. UV–vis absorption spectra of reaction mixtures treated with the Nash's reagent showing the formation of HCHO over (a) CN and (b) WO_3 NPs after a 4 h experiment. Reaction conditions: 20 mg of photocatalyst, 5 mL of 20% vol. MeOH in pure water, 40 °C, static air, 100 W white LED irradiation.

Nash's reagent was prepared by dissolving 15 g of ammonium acetate, 300 μL of acetic acid, and 200 μL of acetylacetone in 100 mL of distilled water.[22]

After MeOH photooxidation experiment, 1 mL of the filtered reaction mixture was added to 1 mL of Nash's reagent and kept on a hotplate at 60 °C for 15 min. After cooling, the UV–vis absorbance spectra were recorded. Under these conditions, HCHO reacts with ammonium acetate and acetylacetone to produce 3,5-diacetyl-1,4-dihydrolutidine, which gives a characteristic absorption peak at 412 nm.

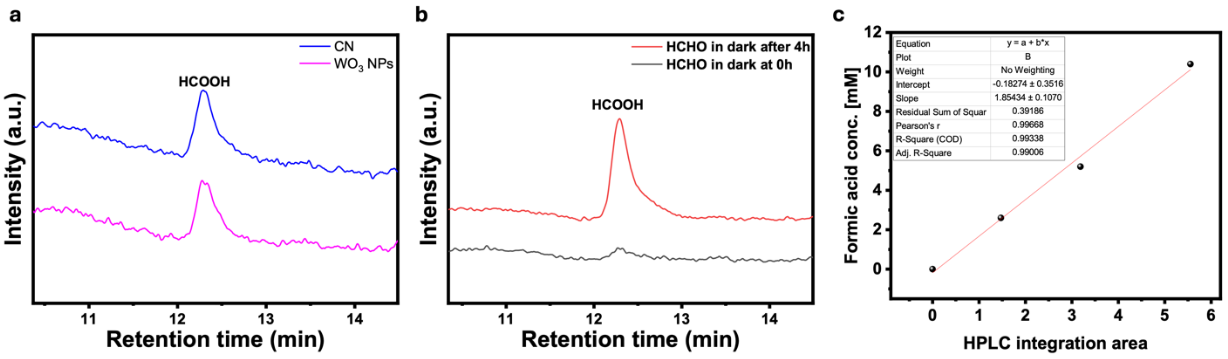

Figure S15. HPLC-DAD spectra obtained for MeOH oxidation experiments. (a) After 4 h illumination using CN (blue), WO_3 NPs (pink) as the photocatalysts, (b) control experiment in the dark without a catalyst, and (c) HPLC HCOOH calibration curves (20% vol. MeOH in 0.08 M NaOH).

The control experiment in the dark was performed by directly taking a mixture of 1 mL of 37% wt. HCHO added to 4 mL of 0.1 M NaOH in a quartz tube and magnetically stirred at 40 °C in the dark. After 4 h of MeOH photooxidation experiment using CN and WO_3 NPs as the photocatalyst or a control experiment in the dark without catalyst, 10 μL of the filtered reaction mixture was diluted to 1 mL using 0.05 M H_2SO_4 solution and injected into the HPLC instrument. The HPLC analysis reveals the formation of ~23 μmol of HCOOH from CN photocatalytic experiment (Figure S15a), which is somewhat higher than the expected yield (17 μmol) from the obtained H_2 yield (8.5 μmol) according to Eq. S1 and Eq. S2. The HPLC analysis of the reaction mixture obtained from dark experiments also shows the formation of HCOOH (Figure S15b), confirming that excess formed or unreacted HCHO can undergo a Cannizzaro reaction in alkaline medium (Eq. S3) at elevated temperatures (40 °C) to produce HCOOH and MeOH. This further supports the excess HCOOH formed during CN photocatalysis experiments.

For WO_3 NPs, we obtained ~22 μmol of HCOOH after 4 h, which is higher than the expected HCOOH yield (10.6 μmol) from the obtained H_2 yield (5.3 μmol). The excess HCOOH in the case of WO_3 NPs, might stem from the deep oxidation of MeOH due to the highly positive valence band of WO_3 or the possible presence of hydroxyl radicals.

MeOH photooxidation reaction:

$$2CH_3OH + 2O_2 \rightarrow 2H_2O_2 + 2HCHO \qquad \text{(Eq. S1)}$$

$$2H_2O_2 + 2HCHO \rightarrow H_2 + 2HCOOH + H_2O_2 \qquad \text{(Eq. S2)}$$

Cannizzaro reaction:

$$2HCHO + HO^- \rightarrow HCOO^- + CH_3OH \qquad \text{(Eq. S3)}$$

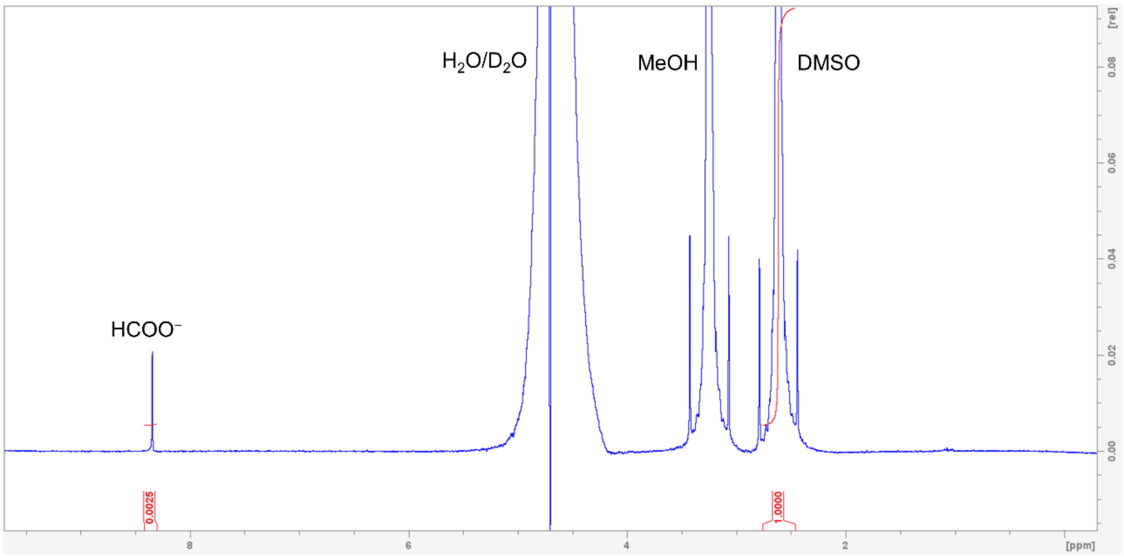

Figure S16. ^{1}H-NMR spectrum for product analysis of MeOH photooxidation in an alkaline medium using CN photocatalysts.

To measure ^{1}H-NMR, first, a 4 h of photocatalytic experiment was performed where 200 μL MeOH were mixed with 5 mL of 0.1 M NaOH and continuously stirred CN powder (15 mg) served as the photocatalyst under illumination. After 4 h, the reaction tube was kept in the dark for several days to ensure complete reaction of the photocatalytically formed H_2O_2 and HCHO to produce the product of the cascade reaction, HCOOH. For the NMR analysis, 100 μL D_2O, 25 μL DMSO, and 875 μL of the filtered reaction mixture were placed in an NMR tube.

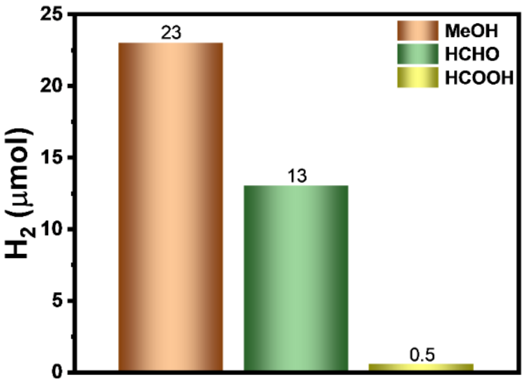

Figure S17. Comparison of H_2 yields obtained after 24 h (photocatalytic experiment using CN) using three different reactants: methanol (brown), formaldehyde (green), and formic acid (yellow).

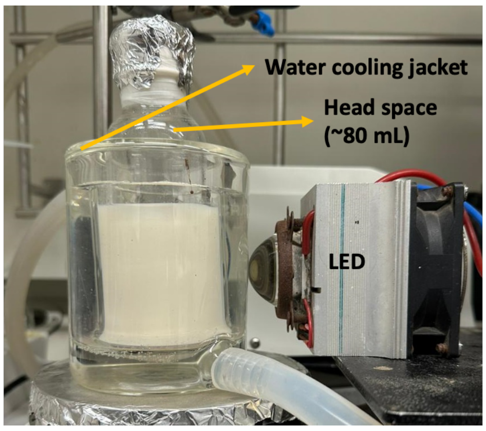

Figure S18. The photocatalytic setup used for scalability experiment. Reaction conditions: 200 mg of CN photocatalyst, 40 °C, static air, 100 W white LED irradiation, 100 mL of 20% vol. MeOH in 0.08 M NaOH.

To demonstrate the scale-up potential, we performed a batch reaction with 200 mg of CN photocatalyst (Figure S18). The reaction yielded 18.5 µmol H_2 after 4 h. It should be noted that the photocatalytically produced H_2O_2 and HCHO (under illumination) react with each other, generating H_2 and HCOOH. This process is independent of light. Therefore, after the illumination was turned off, an additional 7.5 µmol H_2 was measured after 19 h in the dark. The overall H_2 yield was 26 µmol (a total of 23 hours, 4 h under illumination, and 19 h in the dark, when the homogeneous reaction continued).

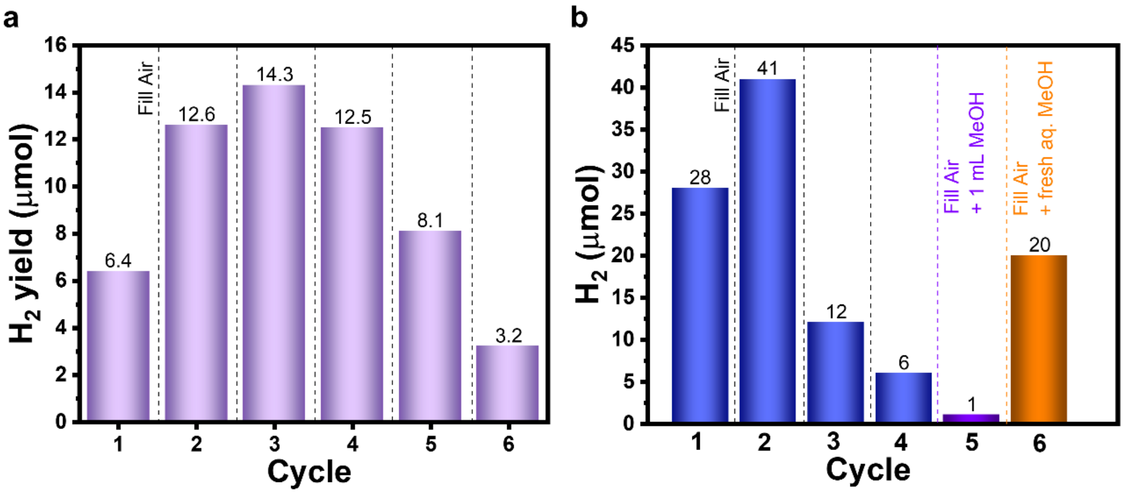

Figure S19. Cycle experiments using CN photocatalyst. (a) 24 h reaction (4 h per cycle) and (b) 6 days reaction (24 h per cycle) with 0.08 M NaOH. After each cycle (indicated by vertical dashed lines) the air reservoir was replenished. Reaction conditions: 20 mg of CN photocatalyst, 40 °C, static air, 100 W white LED irradiation, 5 mL of 20% vol. MeOH in 0.08 M NaOH.

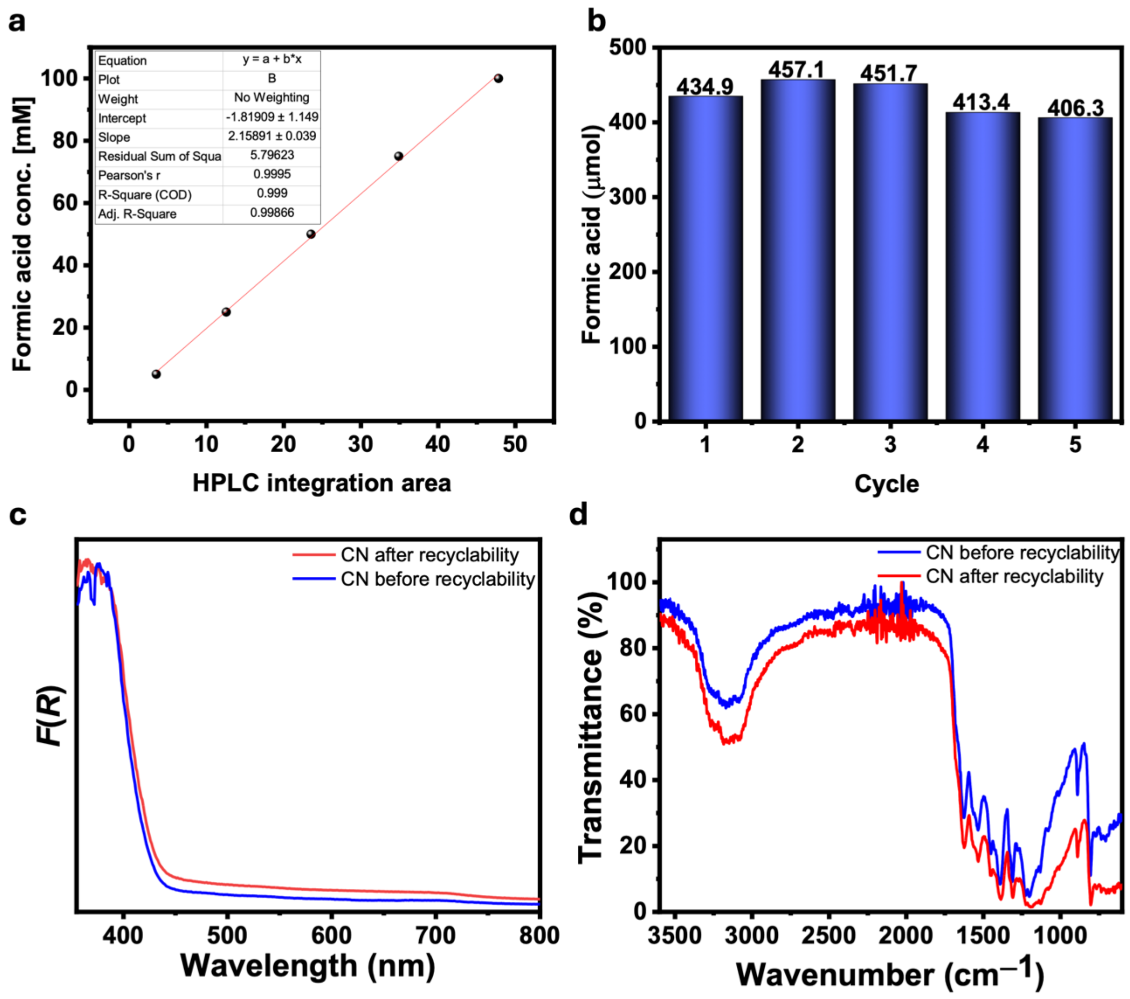

Figure S20. (a) HPLC HCOOH calibration curve (20% vol. MeOH in 0.2 M NaOH), and (b) HCOOH yields obtained in cycle experiments for 10 days (48 h per cycle). Reaction conditions: 20 mg of CN photocatalyst, 5 mL of 20% vol. of MeOH in 0.2 M NaOH, 20 mg CN, static air, 40 °C, 100 W white LED irradiation. After each 48 h cycle, 10 µL of the filtered reaction mixture was diluted to 1 mL using 0.05 M H_2SO_4 solution and injected into the HPLC instrument. (c) DRUV–vis and (d) FTIR spectra of CN powder before and after 10 day photocatalytic recyclability tests.

Table S1. Life cycle inventory to produce the CN photocatalyst, including the Pedigree matrix for unit processes (all distributions assumed to be lognormal).

	Section	Material	Amount	Unit	LCI Database	[a]Pedigree Matrix Uncertainty Factors (A, B, C, D, E)	Comments
**0.81 g of CN**	Input	Melamine	2.23	g	Melamine {GLO}| market for melamine | Cut-off, U	1,4,1,1,1,na	
**Yield 18%**		Cyanuric acid	2.27	g	Created	1,4,1,1,1,na	Based on[23]
		Water	225	g	Water, deionised {RoW}| market for water, deionised | Cut-off, U	1,4,1,1,1,na	
		Nitrogen gas	89	g	Nitrogen, liquid {RoW}| market for nitrogen, liquid | Cut-off, U	2,4,1,1,1,na	Calcination inert gas. Note: In the experimental setup, we used a nitrogen generator rather than the modeled compressed nitrogen; as a result, the reported contributions represent conservative estimate of the contribution of N_2.
		Electricity	40.17	kWh	Electricity, at grid, US/US	4,4,1,1,1,na	
	Emission to air	Ammonia	0.60	g		2,4,5,1,1,na	Approximately 2 NH_3 liberated by melamine pyrolysis at 530 °C for 4 h (mole/mole melamine). Based on[24,25] $C_3H_6N_6(s) \rightarrow C_3N_4(s) + 2NH_3(g)$
		Isocyanic acid	2.27	g		2,4,5,1,1,na	All cyanuric acid is decomposed into iso cyanic acid at 530 °C. Based on[26,27] $C_3H_3N_3O_3(s) \rightarrow 3HNCO(g)$

		Water	225	g		1,4,1,1,1,na	Evaporation
		Nitrogen	89	g		2,4,1,1,1,na	Calcination inert gas
**Cyanuric acid** (for 12.75 g)		Urea	9.2	g	Urea, as 100% $CO(NH_2)_2$ (NPK 46.6-0-0), market mix, at regional storage {RNA} Economic, U	1,4,4,3,1,na	This LCI refers to 1 kg of N, resp. 2.17 kg urea with a N-content of 46%. Thus, to account for the 20 g of urea needed, an amount of 9.2 g is used
Yield 89% based on[23]		Kerosene	40	mL	Kerosene, at refinery/l/US	1,4,4,3,1,na	
		Deionized water	15	g	Water, deionised {RoW}| market for water, deionised | Cut-off, U	1,4,4,3,1,na	
		Electricity	5.4	kWh	Electricity, at grid, US/U2	4,4,1,1,1,na	Used for stirring, heating, drying
	Emission to air	Ammonia	5.67	g		1,4,4,3,1,na	$3CH_4N_2O \rightarrow C_3H_3N_3O_3 + 3NH_3$ Based on[28]
		Water	15	g		1,4,4,3,1,na	Evaporation

[a]The numbers indicate the type of uncertainty considered in the pedigree matrix approach, selected from the options available in the SimaPro uncertainty methodology. A = 1: ‘verified data based on measurements’, A = 2: ‘verified data partly based on assumptions or non-verified data based on measurements’, A = 4: ‘qualified estimate’. B = 4: ‘representative data from only one site relevant for the market considered or some sites but from shorter period’. C = 1: ‘less than 3 years of difference to the time period of the dataset, C = 4: ‘less than 15 years of difference’, and C = 5: ‘age of data unknown or more than 15 years of difference’. D = 1: ‘data from area under study’, D = 3: ‘data from area with similar production conditions’. E = 1: ‘data from enterprises, processes and materials under study’. 'na’ corresponds with unspecified sample size.

Table S2. Life cycle inventory to produce the CN/Pt photocatalyst, including the Pedigree matrix for unit processes (all distributions assumed to be lognormal).

	**Section**	**Material**	**Amount**	**Unit**	**LCI Database**	**[a]Pedigree Matrix Uncertainty Factors (A, B, C, D, E)**	**Comments**
**20 mg of CN/Pt**	Input	Melamine	0.055	g	Melamine {GLO}| market for melamine | Cut-off, U	1,4,1,1,1, na	
**Yield assumed to be 100%**		Cyanuric acid	0.056	g	Created	1,4,1,1,1, na	Based on[23]
		Water	29	g	Water, deionised {RoW}| market for water, deionised | Cut-off, U	1,4,1,1,1, na	
		Nitrogen gas	9.88	g	Nitrogen, liquid {RoW}| market for nitrogen, liquid | Cut-off, U	2,4,1,1,1, na	Calcination inert gas. Note: In the experimental setup, we used a nitrogen generator rather than the modeled compressed nitrogen; as a result, the reported contributions represent conservative estimates.
		Electricity	33.86	kWh	Electricity, at grid, US/US	4,4,1,1,1, na	Used for stirring, sonication, drying, and calcination
		Hexachloroplatinic acid	1.26	mg	Created	1,4,1,1,1, na	Based on[29]
		Methanol	0.791	g	Methanol {GLO}| market for methanol | Cut-off, U	1,4,1,1,1, na	
	Emission to air	Ammonia	0.01485	g		2,4,5,1,1, na	Approximately 2 NH_3 molecules are liberated by melamine pyrolysis at 550 °C for 4 h (mole/mole melamine). Based on[24,25] $C_3H_6N_6(s) \rightarrow C_3N_4(s) + 2NH_3(g)$

		Isocyanic acid	0.056	g		2,4,5,1,1, na	All cyanuric acid is decomposed into cyanic acid at 530 °C. Based on[26] $(CNOH)_3(s) \rightarrow 3HNCO(g)$
		Water	29	g		1,4,1,1,1, na	Evaporation
		Nitrogen	9.88	g		2,4,1,1,1, na	Calcination inert gas
**Hexachloroplatinic acid** (for 1 kg)		Nitric acid	1.2	kg	Nitric acid, in water (60% HNO3) (NPK 13.2-0-0), market mix, at regional storage {RER} Economic, U	1,4,4,3,1, na	Values according to stoichiometry
Based on[29]		Hydrochloric acid	1.8	kg	Hydrochloric acid (30% HCl) (Mannheim), at plant {RER} Economic, U	1,4,4,3,1, na	Values according to stoichiometry
		Platinum extraction	0.37	kg	Platinum {ZA}| platinum group metal, extraction and refinery operations | Cut-off, U	1,4,4,3,1, na	Values according to stoichiometry, 74.2% of platinum stems from South Africa
		Platinum mine operation	0.1	kg	Platinum {RU}| platinum group metal mine operation, ore with high palladium content | Cut-off, U	1,4,4,3,1, na	Values according to stoichiometry, 20.7% of platinum stems from South Africa
		Electricity	0.18	kWh	Electricity, at grid, US/US	4,4,1,1,1, na	Accounts for the energy required to heat up the solution
		Transport (freight train)	1.1	tkm	Transport, freight train {US}| market for transport, freight train | Cut-off, U	1,4,4,3,1, na	Standard distances from ecoinvent guidelines

		Transport (freight lorry)	0.35	tkm	Transport, freight, lorry 16-32 metric ton, EURO6 {RoW}| transport, freight, lorry 16-32 metric ton, EURO6 | Cut-off, U	1,4,4,3,1, na	Standard distances from ecoinvent guidelines
		Organic chemical factory	4e-10	p	Chemical factory, organics {GLO}| market for chemical factory, organics | Cut-off, U	1,4,4,3,1, na	Standard from ecoinvent guidelines
	Emission to air	Nitrogen oxides	0.45	kg		1,4,4,3,1, na	Values according to stoichiometry
		Heat, waste	0.66	MJ		1,4,4,3,1, na	From electricity input
	Emissions to water	Wastewater	0.002	m^3		1,4,4,3,1, na	Water from chemical reactions, values according to stoichiometry

[a]The numbers indicate the type of uncertainty considered in the pedigree matrix approach, selected from the options available in the SimaPro uncertainty methodology. A = 1: ‘verified data based on measurements’, A = 2: ‘verified data partly based on assumptions or non-verified data based on measurements’, A = 4: ‘qualified estimate’. B = 4: ‘representative data from only one site relevant for the market considered or some sites but from shorter period’. C = 1: ‘less than 3 years of difference to the time period of the dataset, C = 4: ‘less than 15 years of difference’, and C = 5: ‘age of data unknown or more than 15 years of difference’. D = 1: ‘data from area under study’, D = 3: ‘data from area with similar production conditions’. E = 1: ‘data from enterprises, processes and materials under study’. 'na' corresponds with unspecified sample size.

Table S3. Life cycle impact assessment results (with standard deviations calculated determined from uncertainty analysis) for producing 1 g CN when the electricity is solar generated.

Impact category	Unit	Total	SD
Ozone depletion	kg CFC-11 eq	5.29E-08	1.02E-08
Global warming	kg CO_2 eq	3.2505302	0.3057129
Smog	kg O_3 eq	0.14474101	0.01301688
Acidification	kg SO_2 eq	0.01915345	0.00159327
Eutrophication	kg N eq	0.00375304	0.00129144
Carcinogenics	CTUh	7.28E-07	7.84E-07
Non carcinogenics	CTUh	4.65E-07	3.58E-05
Respiratory effects	kg PM2.5 eq	0.00188365	0.00024822
Ecotoxicity	CTUe	62.646577	16.596628
Fossil fuel depletion	MJ surplus	4.8812837	0.74289428

Table S4. Life cycle impact assessment results (with standard deviations calculated using uncertainty analysis) for producing 1 g CN and 1 g CN/Pt

Impact category	Unit	1 g CN	SD	1 g CN/Pt	SD
Ozone depletion	kg CFC-11 eq	2.14E-09	2.08E-10	6.05E-08	6.76E-09
Global warming	kg CO_2 eq	39.615315	4.1970393	1321.6325	146.14674
Smog	kg O_3 eq	2.6622404	0.28210401	89.404643	9.8204552
Acidification	kg SO_2 eq	0.34521404	0.03618613	11.515016	1.2610311
Eutrophication	kg N eq	0.00525666	0.00052119	0.18319181	0.01947829
Carcinogenics	CTUh	7.79E-08	1.71E-07	2.85E-06	1.74E-06
Non carcinogenics	CTUh	1.28E-06	8.38E-06	5.44E-05	5.15E-05
Respiratory effects	kg PM2.5 eq	0.01675059	0.00175457	0.55957293	0.0611341
Ecotoxicity	CTUe	16.038583	3.2636192	850.1725	118.45234
Fossil fuel depletion	MJ surplus	25.024269	2.6431657	833.03423	92.044683

Note S1. Renewable energy sources and N_2 recycling offer avenues to reduce the per mass environmental impact

To explore the contribution of energy demand and nitrogen-gas use on the potential reduction in the impacts of the CN synthesis, we closely examined these components. For electricity, the analysis used the "Electricity country mix – Low Voltage – Electricity, at grid, US/US," which represents a mix of multiple energy sources: biomass, coal, petroleum, geothermal, natural gas, nuclear, solar, hydroelectric, and wind.

While energy demand per unit mass of CN and CN/Pt is expected to decrease with production scale-up, energy consumption is likely to remain a dominant factor influencing material synthesis impacts. Alternative sources of energy, such as solar, have the potential to offset the impacts of synthesis energy demand. Considering the growing solar energy infrastructure in Israel (where CN synthesis was carried out, and future work on scale-up for hydrogen generation is planned) and globally, we modeled an LCIA scenario assuming solar-generated electricity as the energy source. The results (Table S3) indicate a substantial reduction in environmental impacts, with the GWP per gram of CN decreasing by nearly 94% (comparing impact values in Table S4 with those in Table S3). Further investigating into the processes contributing to the nitrogen-related impacts revealed that the cryogenic air separation

process used in liquid nitrogen production, along with its associated electricity consumption, accounts for most of the nitrogen's environmental impact. This insight suggests that recycling nitrogen gas during a scaled-up CN synthesis could further reduce its environmental footprint.

(Note: In the experimental system, a nitrogen generator was used, rather than the modeled compressed nitrogen so the contributions presented are conservative.)

Note S2. CO_2-Free Hydrogen Generation via CN Photocatalysis: A Sustainable Alternative to CN/Pt

The overall reaction for hydrogen production over a CN/Pt photocatalyst ('standard' photocatalysis, where Pt serves as the HER co-catalyst, and MeOH as a hole scavenger) is:

$$CH_3OH + H_2O \rightarrow 3H_2 + CO_2$$

The overall reaction for hydrogen production over a CN photocatalyst (via the reported cascade approach) is:

$$2CH_3OH + 2O_2 \rightarrow H_2 + 2\,HCOOH + H_2O_2$$

Table S5. Cost calculation for H_2 production using 'standard' Pt co-catalyst-assisted photocatalysis vs. the reported co-catalyst-free cascade approach.

	[a]H_2 evolution rate (μmol h^{-1} g^{-1})	Estimated H_2 production rate using 1 kg catalyst (L h^{-1})	Amount of Pt needed for 1 kg of catalyst	Pt cost (USD)	Catalyst cost (USD)	Overall cost (USD)	Cost/ L of H_2 h^{-1} (USD)
3% Pt@CN ['standard' photocatalysis]	345	7.7	30 g (3% wt.)	[b]7020	[c]413	7020	965
CN [reported photocatalytic cascade reaction]	107	2.4	—	—	[c]413	413	172

[a]The average H_2 production rate that was experimentally measured for a 4 h reaction is used as a constant rate.

[b]The cost was estimated from the required 8% wt. chloroplatinic acid (H_2PtCl_6) solution to deposit 30 g of Pt on 1 kg of CN.

[c]The costs associated with CN catalyst synthesis.

CN catalyst cost estimation:

The cost of 1 kg of melamine is ~47.05 USD (price taken from the Sigma-Aldrich website, accessed on 22/04/2025, list price of product no. M2659).

The cost of 1 kg of cyanuric acid is ~101 USD (price taken from the Sigma-Aldrich website, accessed on 22/04/2025, list price of product no. 8203581000).

The calculated mass-based yield (%) for CN from MCA is ~18%.

To produce 1 kg of CN, ~5.6 kg of MCA precursor is needed.

The cost for 1 kg of CN would be ~413 USD.

Pt cost estimation:

For 30 g Pt (~0.154 mol), ~63.02 g of H_2PtCl_6 is required.

For 63.02 g of chloroplatinic acid, ~788 g or 750 mL of 8% wt. chloroplatinic acid (H_2PtCl_6) solution is required.

The cost of 250 mL of 8% wt. chloroplatinic acid (H_2PtCl_6) solution is ~2340 USD (price taken from the Sigma-Aldrich website, accessed on 22/04/2025, list price of product no. 262587).

Relative cost of operation:

Normalizing the photocatalyst synthesis cost to relevant H_2 production rate (assumed constant; in units of L h^{-1}) shows that the proposed co-catalyst-free photocatalytic cascade approach using CN is ~5.6 times cheaper than the traditional Pt-decorated CN photocatalysis (172 vs. 965 USD h L_{H2}^{-1}).

Supporting Information References

(1) Shalom, M.; Gimenez, S.; Schipper, F.; Herraiz-Cardona, I.; Bisquert, J.; Antonietti, M. Controlled Carbon Nitride Growth on Surfaces for Hydrogen Evolution Electrodes. *Angew. Chem. Int. Ed.* **2014**, *53* (14), 3654–3658. https://doi.org/https://doi.org/10.1002/anie.201309415.

(2) Wei, Z.; Liu, M.; Zhang, Z.; Yao, W.; Tan, H.; Zhu, Y. Efficient Visible-Light-Driven Selective Oxygen Reduction to Hydrogen Peroxide by Oxygen-Enriched Graphitic Carbon Nitride Polymers. *Energy Environ. Sci.* **2018**, *11* (9), 2581–2589. https://doi.org/10.1039/C8EE01316K.

(3) Kresse, G.; Furthmüller, J. Efficiency of Ab-Initio Total Energy Calculations for Metals and Semiconductors Using a Plane-Wave Basis Set. *Comput. Mater. Sci.* **1996**, *6* (1), 15–50. https://doi.org/10.1016/0927-0256(96)00008-0.

(4) Nørskov, J. K.; Rossmeisl, J.; Logadottir, A.; Lindqvist, L.; Kitchin, J. R.; Bligaard, T.; Jónsson, H. Origin of the Overpotential for Oxygen Reduction at a Fuel-Cell Cathode. *J. Phys. Chem. B* **2004**, *108* (46), 17886–17892. https://doi.org/10.1021/jp047349j.

(5) Perdew, J. P.; Burke, K.; Ernzerhof, M. Generalized Gradient Approximation Made Simple. *Phys. Rev. Lett.* **1996**, *77* (18), 3865–3868. https://doi.org/10.1103/PhysRevLett.77.3865.

(6) Blöchl, P. E. Projector Augmented-Wave Method. *Phys. Rev. B* **1994**, *50* (24), 17953–17979. https://doi.org/10.1103/PhysRevB.50.17953.

(7) Pracht, P.; Grimme, S.; Bannwarth, C.; Bohle, F.; Ehlert, S.; Feldmann, G.; Gorges, J.; Müller, M.; Neudecker, T.; Plett, C.; Spicher, S.; Steinbach, P.; Wesołowski, P. A.; Zeller, F. CREST—A Program for the Exploration of Low-Energy Molecular Chemical Space. *J. Chem. Phys.* **2024**, *160* (11), 114110. https://doi.org/10.1063/5.0197592.

(8) Bannwarth, C.; Ehlert, S.; Grimme, S. GFN2-XTB—An Accurate and Broadly Parametrized Self-Consistent Tight-Binding Quantum Chemical Method with Multipole Electrostatics and Density-Dependent Dispersion Contributions. *J. Chem. Theory Comput.* **2019**, *15* (3), 1652–1671. https://doi.org/10.1021/acs.jctc.8b01176.

(9) Frisch, M. J.; Trucks, G. W.; Schlegel, H. B.; Scuseria, G. E.; Robb, M. A.; Cheeseman, J. R.; Scalmani, G.; Barone, V.; Mennucci, B.; Petersson, G. A. Bréch 09 Revision A. 02. *Gaussian Inc., Wallingford CT* **2016**.

(10) Stephens, P. J.; Devlin, F. J.; Chabalowski, C. F.; Frisch, M. J. Ab Initio Calculation of Vibrational Absorption and Circular Dichroism Spectra Using Density Functional Force Fields. *J. Phys. Chem.* **1994**, *98* (45), 11623–11627. https://doi.org/10.1021/j100096a001.

(11) Lee, C.; Yang, W.; Parr, R. G. Development of the Colle-Salvetti Correlation-Energy Formula into a Functional of the Electron Density. *Phys. Rev. B* **1988**, *37* (2), 785–789. https://doi.org/10.1103/PhysRevB.37.785.

(12) Becke, A. D. Density-functional Thermochemistry. III. The Role of Exact Exchange. *J. Chem. Phys.* **1993**, *98* (7), 5648–5652. https://doi.org/10.1063/1.464913.

(13) Hehre, W. J.; Ditchfield, R.; Pople, J. A. Self—Consistent Molecular Orbital Methods. XII. Further Extensions of Gaussian—Type Basis Sets for Use in Molecular Orbital Studies of Organic Molecules. *J. Chem. Phys.* **1972**, *56* (5), 2257–2261. https://doi.org/10.1063/1.1677527.

(14) Hariharan, P. C.; Pople, J. A. The Influence of Polarization Functions on Molecular Orbital Hydrogenation Energies. *Theor. Chim. Acta* **1973**, *28* (3), 213–222. https://doi.org/10.1007/BF00533485.

(15) Ditchfield, R.; Hehre, W. J.; Pople, J. A. Self-Consistent Molecular-Orbital Methods. IX. An Extended Gaussian-Type Basis for Molecular-Orbital Studies of Organic Molecules. *J. Chem. Phys.* **1971**, *54* (2), 724–728. https://doi.org/10.1063/1.1674902.

(16) *U.S. Life Cycle Inventory Database*. U.S. Life Cycle Inventory Database. https://www.nrel.gov/analysis/lci.html (accessed 2024-11-24).

(17) Simapro. *Industry data LCA library*. https://simapro.com/products/industry-data/ (accessed 2025-05-01).

(18) Wernet, G.; Bauer, C.; Steubing, B.; Reinhard, J.; Moreno-Ruiz, E.; Weidema, B. The Ecoinvent Database Version 3 (Part I): Overview and Methodology. *Int. J. Life Cycle Assess.* **2016**, *21* (9), 1218–1230. https://doi.org/10.1007/s11367-016-1087-8.

(19) *Long Trail Sustainability – Sustainability Software, Training and Consulting*. https://longtrailsustainability.com (accessed 2024-11-24).

(20) Bare, J. *Tool for Reduction and Assessment of Chemicals and Other Environmental Impacts (TRACI)*. Traci 2.1. https://www.epa.gov/chemical-research/tool-reduction-and-assessment-chemicals-and-other-environmental-impacts-traci (accessed 2024-11-24).

(21) Bare, J. C.; Norris, G. A.; Pennington, D. W.; McKone, T. TRACI: The Tool for the Reduction and Assessment of Chemical and Other Environmental Impacts. *J. Ind. Ecol.* **2003**, *6* (3–4), 49–78. https://doi.org/10.1162/108819802766269539.

(22) Low, Lai Kim; Ng, C. S. Determination of Chemical Properties of Meat: Determination of Formaldehyde in Fish Meat Using Nash's Reagent. *Lab. Man. Anal. Methods Proced. Fish Fish Prod.* **1987**, B-5.1.

(23) She, D.-M.; Yu, H.-L.; Huang, Q.-L.; Li, F.-M.; Li, C.-J. Liquid-Phase Synthesis of Cyanuric Acid from Urea. *Molecules*. 2010, pp 1898–1902. https://doi.org/10.3390/molecules15031898.

(24) Li, X.; Zhang, J.; Shen, L.; Ma, Y.; Lei, W.; Cui, Q.; Zou, G. Preparation and Characterization of Graphitic Carbon Nitride through Pyrolysis of Melamine. *Appl. Phys. A* **2009**, *94* (2), 387–392. https://doi.org/10.1007/s00339-008-4816-4.

(25) May, H. Pyrolysis of Melamine. *J. Appl. Chem.* **1959**, *9* (6), 340–344. https://doi.org/10.1002/jctb.5010090608.

(26) Huthmacher, K.; Most, D. Cyanuric Acid and Cyanuric Chloride. In *Ullmann's Encyclopedia of Industrial Chemistry*; 2000. https://doi.org/10.1002/14356007.a08_191.

(27) Schaber, P. M.; Colson, J.; Higgins, S.; Thielen, D.; Anspach, B.; Brauer, J. Thermal Decomposition (Pyrolysis) of Urea in an Open Reaction Vessel. *Thermochim. Acta*

2004, *424* (1–2), 131–142. https://doi.org/10.1016/j.tca.2004.05.018.

(28) Wu, F.; Zhou, Z.; Hicks, A. L. Life Cycle Impact of Titanium Dioxide Nanoparticle Synthesis through Physical, Chemical, and Biological Routes. *Environ. Sci. Technol.* **2019**, *53* (8), 4078–4087. https://doi.org/10.1021/acs.est.8b06800.

(29) Notter, D. A.; Kouravelou, K.; Karachalios, T.; Daletou, M. K.; Haberland, N. T. Life Cycle Assessment of PEM FC Applications: Electric Mobility and μ-CHP. *Energy Environ. Sci.* **2015**, *8* (7), 1969–1985. https://doi.org/10.1039/C5EE01082A.
